# Cancer mortality by country of birth and cancer type in Sweden: A 25‐year registry‐based cohort study

**DOI:** 10.1002/cam4.70020

**Published:** 2024-07-17

**Authors:** Daniel Nigusse Tollosa, Kazem Zendehdel, Alessandro Procopio, Agneta Cederström, Paolo Boffetta, Eero Pukkala, Mikael Rostila

**Affiliations:** ^1^ Department of Public Health Sciences Stockholm University Stockholm Sweden; ^2^ Cancer Research Center, Cancer Institute Tehran University of Medical Sciences Tehran Iran; ^3^ Department of Medical and Surgical Sciences University of Bologna Bologna Italy; ^4^ Stony Brook Cancer Center Stony Brook University Stony Brook New York USA; ^5^ Finnish Cancer Registry Institute for Statistical and Epidemiological Cancer Research Helsinki Finland; ^6^ Health Sciences Unit, Faculty of Social Sciences Tampere University Tampere Finland; ^7^ Centre for Health Equity Studies (CHESS) Stockholm University/Karolinska Institutet Stockholm Sweden; ^8^ Aging Research Center (ARC) Karolinska Institutet Solna Sweden

**Keywords:** cancer, immigrants, mortality, registered‐based, Sweden

## Abstract

Numerous studies have reported lower overall cancer mortality rates among immigrants compared to native populations. However, limited information exists regarding cancer mortality among immigrants based on specific birth countries and cancer types. We used population‐based registries and followed 10 million individuals aged 20 years or older in Sweden between 1992 and 2016. The Cox proportional hazard model was used to explore the disparities in cancer mortality by country of birth and cancer type, stratified by gender. Age‐standardized mortality rates were also computed using the world standard population. Hazard ratio (HR) of all‐site cancer was slightly lower among immigrants (males: HR_m_ = 0.97: 95% confidence interval: 0.95, 0.98; females: HR_f_ = 0.93: 0.91, 0.94) than Swedish‐born population. However, the immigrants showed higher mortality for infection‐related cancers, including liver (HR_f_ = 1.10: 1.01, 1.19; HR_m_ = 1.10: 1.02, 1.17), stomach (HR_f_ = 1.39: 1.31, 1.49; HR_m_ = 1.33: 1.26, 1.41) cancers, and tobacco‐related cancers, including lung (HR_m_ = 1.44: 1.40, 1.49), and laryngeal cancers (HR_m_ = 1.47: 1.24, 1.75). The HR of mesothelioma was also significantly higher in immigrants (HR_f_ = 1.44: 1.10, 1.90). Mortality from lung cancer was specifically higher in men from Nordic (HR_m_ = 1.41: 1.27, 1.55) and non‐Nordic Europe (HR_m_ = 1.49: 1.43, 1.55) countries and lower in Asian (HR_m_ = 0.78: 0.66, 0.93) and South American men (HR_m_ = 0.70: 0.57, 0.87). In conclusion, there are large variations in cancer mortality by country of birth, and cancer type and require regular surveillance. Our detailed analyses lead to some novel findings such as excess mortality rate of mesothelioma and laryngeal cancers in Immigrants in Sweden. A targeted cancer prevention program among immigrants in Sweden is needed.

## 1 INTRODUCTION

Cancer poses a major global public health problem, accounting for nearly 10 million deaths in 2020,^1^ and it is the second most common cause of death in the Swedish population, with nearly 24,000 deaths in 2021.^2^ Sweden's growing and aging immigrant population, now constituting approximately 20% of the total population, underscores the importance of prioritizing cancer research and health care for immigrants.^3^

Research in the field of migration and health has found that most migrant groups live longer than the non‐immigrant population despite occupying lower socioeconomic positions, a phenomenon known as the “healthy immigrant paradox”.^4^ Accordingly, previous studies examining the rate of cancer mortality among immigrants in Sweden have predominantly shown that most migrant groups, with the exception of immigrants from the Nordic region, experience lower all‐cause mortality rates compared to the non‐immigrant population.^5^, ^6^, ^7^, ^8^, ^9^, ^10^, ^11^, ^12^

The risk of dying due to malignancies is higher in males, as compared to females, for a majority of cancer types^13^ Socioeconomic status also plays a significant role in cancer mortality disparities.^7^ Immigrants with a lower socioeconomic position (SEP) may have limited access to preventive healthcare services, early cancer detection, and high‐quality cancer treatment.^14^ Health risk behaviors (e.g., smoking and alcohol consumption) and environmental risks (poor housing, air pollution, work‐related health hazards) related to a higher risk of cancer mortality might also be higher among immigrants with low SEP.^15^

Most of studies on cancer among immigrants have primarily focused on the incidence and mortality disparities in all cancer sites^7^, ^12^ or in more common cancer sites.^8^, ^10^, ^11^, ^16^ In addition, they have examined the disparities across immigrants by nativity (immigrants vs. non‐immigrants) or broad groups of immigrants according to their region of birth.^11^, ^16^, ^17^ As the immigrant population grows in size and the person‐time and age of the immigrants increase, it would be possible to investigate the disparities for rare cancers and at the country of origin. Moreover, the composition of the immigrant population is evolving, marked by distinct reasons for immigration and diverse country origins among more recent immigrants compared to those in previous decades. This shift makes it challenging to generalize previous findings to this new cohort of immigrants. Consequently, there is a noticeable gap in comprehensive studies that explore cancer mortality by country of birth, as highlighted by a recent systematic review on all‐cause mortality rates in the Nordic countries.^18^

This study took advantage of the Swedish high‐quality national registries to examine disparities in cancer mortality by country of birth, gender, and specific cancer type, while also considering confounding by socio‐demographic factors. Such comprehensive knowledge is pivotal for the formation of health policies and preventive action aiming to reduce the cancer burden in immigrants.

## 2 MATERIALS AND METHODS

### 2.1 Data sources

This study utilized registry data linkage from three sources, including (1) The Cause of Death Register provided data on the date of death, and underlying and contributing causes of death using ICD‐9 codes from 1992 to 1996 and ICD‐10 codes from 1997 onwards.^19^ The quality of the cause of death register is generally considered as high and being a valuable resource for public health research and policy;^20^ (2) The Longitudinal Database of Health, Insurance, and Labor Market Studies (LISA) maintained by Statistics Sweden compiles annual data covering the Swedish population aged ≥16 years registered on December 31 each year since 1990 (since 2010 individuals aged 15 years included). We used this registry to obtain information on socioeconomic factors including education, disposable income, and civil status;^21^ (3) The Total Population Registry is also maintained by Statistics Sweden and provides information on the entire population's dates of birth, death, sex, in and out‐migration, and country of birth.^22^ These datasets were merged using a unique 10‐digit personal identity number, which was then replaced by a serial number to ensure anonymity. The study did not require informed consent from participants and was approved by the Swedish Ethical Review Authority (decision 1: 2017/716‐31 and decision 2: 2023‐02550‐02).

### 2.2 Cohort

**FIGURE 1:**
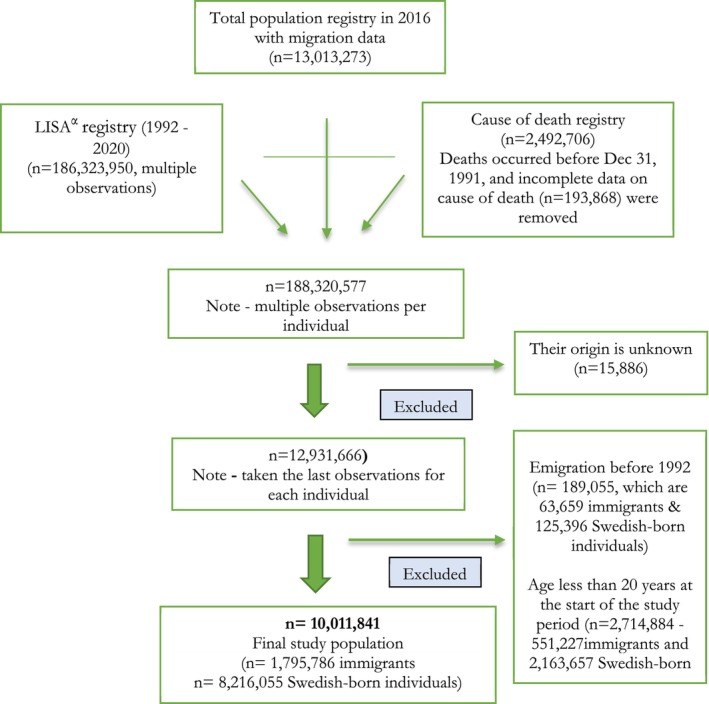
Final study population and exclusion criteria, Sweden, 1992 to 2016. ^α^The Longitudinal Database of Health, Insurance, and Labor Market Studies.

The study participants were enrolled using an open‐cohort design, for all individuals who had been residing in Sweden since January 1, 1992, and aged 20 years or older. Participants were followed up until they emigrated, died, or until the end of the study period, that is, on December 31, 2016, whichever came first. Individuals with incomplete data on the country of birth and those who had moved out of Sweden before 1992 were excluded from the study. Figure 1 shows the selection of the study cohort and the number of excluded participants with their respective origins.

### 2.3 Variables (outcomes and covariates)

The person‐years, measured in days, was determined by calculating the duration between the start and exit of the follow‐up. For immigrants, the follow‐up began 2 years after their arrival in Sweden. This adjustment was made to reduce the likelihood of including those immigrants who arrived in Sweden nearly at the end‐of‐life stage with cancer. This commencement date was either January 01, 1992, for those arriving in Sweden before January 01, 1990, or 2 years after for those who arrived in Sweden after January 01, 1990. For individuals born in Sweden, the follow‐up commenced on January 01, 1992. Immigrants were stratified into the largest immigrant groups in Sweden to ensure a sufficiently large analytical sample size: Nordic (Finland, Norway, Denmark), Non‐Nordic Europe (Turkey, Germany, Austria, Netherlands, France, former Yugoslavia, Bosnia‐Hercegovina, Italy, Spain, Greece, Croatia, Poland, Hungary, former Czechoslovakia, Romania, former Soviet Union, Russia, Estonia, UK and North Ireland), North America (USA), South America (Chile), Africa (Ethiopia, Somalia), Middle East (Iraq, Syria, Lebanon, Iran), and Asia (India, Vietnam, Thailand, and China).

The mortality rates were estimated for all cancers combined and separately for 16 cancer types in both sexes (lung, colorectal, pancreas, liver, stomach, brain and CNS, kidney, hematological (leukemia, Hodgkin lymphoma, non‐Hodgkin lymphoma, and multiple myeloma), head and neck (oropharyngeal, hypopharyngeal, nasopharyngeal, lip, and oral), bladder, esophageal, melanoma, gall bladder, thyroid, mesothelioma, and laryngeal), four cancers specific to females (cervical, ovarian, corpus uteri, vulva, and vaginal), and prostate cancer in males. In the case of breast cancer, the analysis was restricted to women because of the small number of events among men.

To account for potential confounding factors for disparities of cancer mortality rate ratios between immigrants and non‐immigrants, covariates including attained age, education (grouped into primary education, secondary education, post‐secondary education or tertiary education, and unknown/missing), disposable income (categorized into five groups based on quintiles), marital status (single, married, divorced, or widowed), and calendar period (with 5 years interval) were utilized for adjustment. All socioeconomic characteristics were observed at the exit date of the study.

### 2.4 Statistical methods

We calculated the age‐standardized mortality rate (males: ASMR_m_, females: ASMR_f_) per 100,000 person‐years using the world standard population for all sites combined and 22 specific cancer types. ASMRs for all cancer sites combined are reported in this publication, and the results for specific cancer types are available in Tables S1 and S2.

The Cox proportional hazard model, using age as the underlying time scale, was employed to determine the relative risk of mortality, represented as hazard ratios (males: HR_m_, females: HR_f_) and corresponding 95% confidence intervals (CI), in immigrant populations by country of birth. In all estimates, the reference group for comparison was individuals born in Sweden. The HRs of cancer mortality by country of birth were estimated for three aggregated levels of immigrants—all immigrants together, immigrants grouped based on region and specific country of birth. HRs were only reported for countries with more than at least five deaths reported during the follow‐up. We used forest plots to visualize the HRs on a log scale for the top 9 common cancer types in both genders. However, results for all other cancer types are provided as Data S1. Two Cox regression models were constructed to evaluate the HRs by cancer type for all immigrant groups. Model 1 adjusted only for age, while Model 2 adjusted for all covariates—age, income, education, marital status, and calendar year. A *p*‐value of <0.05 was used to declare statistical significance.

All analyses were stratified by gender and performed using Stata software (Stata Ver.17, Stata Corp, College Station, Texas 77,845 USA).

## 3 RESULTS

The study cohort included 10,011,841 individuals, of which 18% were foreign‐born individuals and 50.2% were female (Table 1). A higher proportion of immigrants were either married or divorced, had completed tertiary education, and were younger at entry of the follow‐up compared to Swedish‐born individuals. A higher percentage of the immigrants had very low disposable income compared with non‐immigrants. Across the calendar periods, the proportion of cancer deaths is relatively constant in Swedish‐born individuals whereas on average increased by 0.4% in immigrants annually (Table 1).

**TABLE 1:** **—**Characteristics of participants in the study by gender and origin for all‐site cancer mortality, Sweden, 1992 to 2016.

Characteristics	Male				Female			
	Foreign‐born (*n* = 905,472)		Sweden‐born (*n* = 4,139,762)		Foreign‐born (*n* = 890,314)		Sweden‐born (*n* = 4,076,293)	
Age (in years) at entry (Mean, SD)	36.4 (14.1)	–	39.7 (19.7)	–	37.8 (15.9)	‐	42.3 (21.4)	–
Age (in years) at exit (Mean, SD)	45.81 (17.49)	–	56.92 (20.67)	–	50.2 (19.3)	‐	59.2 (22.2)	–
	Cases (person‐years in 100,000)	Freq (%)	Cases (person‐years in 100,000)	Freq (%)	Cases (person‐years in 100,000)	Freq (%)	Cases (person‐years in 100,000)	Freq (%)
Age groups (at exit)	*P* < 0.01				*P* < 0.01			
>20 to <40	491 (22.5)	374,037 (42.0)	2695 (111.6)	1,095,999 (26.9)	589 (22.2)	348,147 (38.5)	3217 (104.3)	1,034,759 (25.0)
> = 40 to <60	5076 (43.0)	294,850 (33.1)	27,960 (255.9)	1,110,143 (27.2)	5040 (44.4)	286,480 (31.6)	32,788 (241.2)	1,030,238 (24.9)
> = 60 to <80	16,032 (33.1)	178,386 (20.1)	142,850 (253.2)	1,256,310 (30.8)	14,433 (36.5)	188,092 (20.8)	116,378 (245.9)	1,154,540 (27.9)
> = 80	4579 (7.8)	43,041 (4.8)	78,004 (96.9)	613,841 (15.1)	5929 (15.0)	82,753 (9.1)	76,917 (146.3)	920,225 (22.2)
Education	*P* < 0.01				*P* < 0.01			
Primary completed	9720 (26.2)	194,662 (22.6)	119,749 (182.6)	1,040,237 (25.8)	10,652 (32.0)	217,249 (24.1)	109,910 (178.8)	998,599 (24.4)
Secondary completed	9823 (48.9)	350,629 (39.6)	79,523 (365.8)	2,021,993 (50.2)	8397 (47.8)	323,918 (35.9))	68,100 (324.5)	1,752,329 (42.8)
Tertiary completed	3480 (26.8)	248,492 (28.1)	28,209 (160.6)	850,238 (21.1)	3198 (32.5)	278,552 (30.9)	25,550 (219.4)	1,137,175 (27.8)
Not known	2414 (4.5)	90,952 (10.3)	13,977 (8.3)	119,503 (3.0)	3041 (5.7))	80,931 (9.0)	16,632 (14.8)	208,301 (5.1)
Individual disposable income	*P* < 0.01				*P* < 0.01			
Very Low	7710 (20.1)	264,813 (29.9)	67,089 (58.3)	591,493 (14.7)	11,159 (23.1)	285,105 (31.7)	100,068 (95.2)	83,8267 (20.5)
Low	9450 (20.5)	145,702 (16.4)	84,194 (114.8)	694,340 (17.20)	8747 (31.2)	192,881 (21.4)	70,427 (183.2)	951,503 (23.2)
Middle	4799 (21.7)	159,197 (17.9)	49,725 (148.4)	785,473 (19.50)	2923 (24.6)	172,737 (19.2)	27,052 (161.8)	865,433 (21.1)
High	1462 (21.9)	161,999 (18.3)	17,188 (160.7)	848,546 (21.1)	810 (23.1)	150,399 (16.7)	8308 (164.9)	821,872 (20.1)
Very high	2016 (22.2)	153,024 (17.3)	23,262 (235.2)	1,112,119 (27.6)	1649 (16.1)	99,498 (11.1)	14,337 (132.4)	619,329 (15.1)
Marital status	*P* < 0.01				*P* < 0.01			
Single	2629 (24.6)	290,108 (32.8)	31,419 (225.9)	1,553,793 (38.5)	1901 (17.8)	201,252 (22.4)	23,239 (177.4)	1,275,381 (31.1)
Married	15,348 (56.5)	433,443 (49.0)	140,932 (352.4)	1,746,711 (43.3)	10,156 (54.8)	426,397 (47.4)	81,051 (308.6)	1,463,997 (35.8)
Divorced	4892 (21.1)	136,785 (15.5)	30,108 (90.6)	431,170 (10.7)	5414 (28.2)	173,300 (19.3)	31,126 (108.1)	501,006 (12.2)
Widowed	2563 (4.1)	24,003 (2.7)	38,982 (48.3)	299,681 (7.4)	7816 (17.2)	99,467 (11.1)	84,772 (143.2)	855,255 (20.9)
Calendar year	*P* < 0.01				*P* < 0.01			
1992–1996	3686 (16.2)	‐	50,387 (141.9)	–	3828 (17.8)	‐	46,175 (148.3)	–
1997–2002	4471 (18.1)	‐	50,615 (142.0)	–	4547 (20.4)	‐	45,563 (147.5)	–
2002–2006	5252 (20.6)	‐	50,597 (142.1)	–	5249 (22.6)	‐	45,975 (146.3)	–
2007–2011	5968 (23.6)	‐	49,581 (144.1)	–	5757 (26.2)	‐	45,562 (146.8)	–
2012–2016	6801 (28.4)	‐	50,329 (147.5)	–	6610 (31.0)	‐	46,025 (148.8)	–

### 3.1 All‐cancermortality rates

A total of 532,873 cancer deaths were reported in Sweden during the study period, from which 52,168 deaths were registered for the immigrant population. The ASMRs for all cancer types combined were 81.8 and 113.7 per 100,000 person‐time in female and male immigrants, respectively. The rates were lower in females but higher in male immigrants than the Swedish‐born population (ASMR_f_ = 86.4; ASMR_m_ = 106.4 per 100,000).

Compared to Swedish‐born individuals, HRs were somewhat lower both in immigrant women (HR_f_ = 0.93, 95% CI: 0.91, 0.94) and men (HR_m_ = 0.97: 0.95, 0.98), and also among immigrants from non‐Nordic European countries, North America, South America, Africa, Middle East, and Asia. However, male immigrants from the Nordic countries had 11% higher overall cancer mortality rates compared to the Swedish‐born population (HR_m_ = 1.11: 1.09, 1.13). The highest age‐standardized cancer mortality rate was observed among immigrants from the Nordic region (ASMR_f_ = 91.3; ASMR_m_ = 125.) followed by non‐Nordic Europe (ASMR_f_ = 80.5; ASMR_m_ = 114.4). The lowest rate was observed in immigrants from the Middle East (ASMR_f_ = 52.3; ASMRm = 79.1). Stratified estimates by calendar period show that mortality rates are generally been on a downward trend over the past decades for both immigrant groups and Sweden‐born. By age groups, Nordic immigrants have shown the highest rates of cancer deaths, mainly in the mid and older age (Figures S1 and S2).

At the country level, we found that immigrants from Denmark (HR_f_ = 1.17: 1.12, 1.23; HR_m_ = 1.15: 1.10, 1.19), Norway (HR_f_ = 1.04: 1.00, 1.08; HR_m_ = 1.04: 1.00, 1.09), and male immigrants from Finland (HR_m_ = 1.11: 1.09, 1.14) had significantly higher risk of all‐site cancer deaths compared to the non‐immigrants in Sweden. Except for Bosnian‐Hercegovina and Estonia female (HR_f_ = 1.17: 1.09, 1.25; HR_f_ = 1.08: 1.00, 1.16, respectively) and male immigrants from former Yugoslavia (HR_m_ = 1.06: 1.01, 1.11), Bosnia‐Hercegovina (HR_m_ = 1.13: 1.06, 1.21), and Estonia (HR_m_ = 1.13: 1.06, 1.21) who had significantly higher risk of death due to all cancer types combined, most of other immigrants had a significantly lower risk of all‐site cancer mortality compared to Swedish‐born individuals, with the Middle East and Asia group being in the lowest rank (Table 2). The highest ASMR was observed among male immigrants from Bosnia‐Hercegovina (ASMR = 142.7).

**TABLE 2:** —Number of cancer deaths, person‐time, age‐standardized mortality rates (ASMR) per 100,000 person‐years, and adjusted hazard ration with 95% confidence intervals for all‐site cancer mortality among immigrants in Sweden, 1992 to 2016.

Regions/countries	Male						Female					
	Deaths	Person‐years per 100,000	ASMR (per 100,000)	HR, 95% CI			Deaths	Person‐years per 100,000	ASMR (per 100,000)	HR, 95% CI		
Sweden (ref)	251,509	717.6	106.4	1.00			229,300	717.6	86.4	1.00		
All immigrants	26,178	106.4	113.7	0.97	0.95	0.98***	25,990	106.4	81.8	0.93	0.91	0.94***
Nordic												
Finland	6696	16.3	123.6	1.11	1.09	1.14***	8458	24.4	86.4	0.97	0.95	0.99***
Denmark	2587	4.2	135.1	1.15	1.10	1.19***	2013	4.0	113	1.17	1.12	1.23***
Norway	1921	3.4	124.3	1.04	0.99	1.09	2609	5.2	96.7	1.04	0.99	1.08
All Nordic	11,247	24.2	125.9	1.11	1.09	1.13***	13,134	33.8	91.3	1.01	0.99	1.03
Non‐Nordic Europe												
Turkey	509	4.0	94.1	0.70	0.64	0.76***	361	3.5	58.1	0.63	0.57	0.70***
Germany	1789	3.8	108.1	0.98	0.93	1.02	2122	4.87	81.7	0.95	0.91	0.99*
Austria	358	0.7	106.6	0.99	0.89	1.09	238	0.6	78.9	0.96	0.84	1.09
Netherlands	218	0.6	121.0	1.02	0.89	1.16	114	0.4	83.5	0.83	0.69	1.00
France	127	0.6	111.9	0.97	0.82	1.16	86	0.5	70	0.85	0.69	1.06
Former Yugoslavia	1772	7.1	124.6	1.06	1.01	1.11*	1168	6.9	77.5	0.91	0.85	0.96**
Bosnian‐Hercegovina	990	4.8	142.7	1.13	1.06	1.21***	835	4.9	91.4	1.17	1.09	1.25***
Italy	423	0.9	124.6	1.08	0.98	1.19	140	0.5	88.6	0.99	0.83	1.17
Spain	155	0.6	104.6	0.92	0.78	1.07	69	0.4	64	0.71	0.56	0.90**
Greece	316	1.4	95.5	0.76	0.68	0.85***	133	0.9	57.5	0.61	0.52	0.73***
Croatia	107	0.6	92.6	0.78	0.65	0.95*	79	0.6	62.6	0.76	0.61	0.95*
Poland	873	3.9	109.3	0.88	0.82	0.94***	1360	6.7	83.2	0.92	0.87	0.97**
Hungary	805	1.6	122.5	1.05	0.98	1.13	529	1.6	87.6	1.03	0.94	1.12
Former Czechoslovakia	363	0.7	115.8	0.93	0.84	1.04	309	0.9	83.4	0.99	0.88	1.10
Romania	263	1.4	101.2	0.75	0.66	0.85***	303	1.6	81.3	0.96	0.86	1.08
Former Sovjetunion	300	0.6	106.2	0.82	0.72	0.92**	355	1.0	76.8	0.88	0.79	0.98*
Russia	42	0.4	102.1	0.82	0.61	1.11	100	1.2	64.3	0.74	0.61	0.90**
Estonia	982	1.0	121.0	1.13	1.06	1.21***	817	1.3	82.8	1.08	1.00	1.16
UK and North Ireland	313	2.0	97.1	0.82	0.73	0.91***	227	1.0	86.7	0.97	0.85	1.10
All Non‐Nordic Europe	11,282	40.	114.4	0.96	0.94	0.98***	9833	42.9	80.5	0.93	0.91	0.95**
North America												
USA	485	1.4	107.0	0.94	0.86	1.03	405	1.3	82	0.87	0.79	0.96**
All North America	590	2.4	101.4	0.91	0.84	0.98**	492	2.3	75.2	0.82	0.75	0.90**
South America												
Chile	312	2.8	94.9	0.70	0.63	0.79***	316	2.8	64.6	0.69	0.62	0.77***
All South America	501	5.0	92.0	0.70	0.64	0.77***	507	5.3	62.1	0.67	0.61	0.73***
Africa												
Ethiopia	74	1.2	94.7	0.84	0.67	1.05	63	1.1	70	0.91	0.71	1.17
Somalia	65	1.8	69.5	0.60	0.47	0.76***	62	1.7	42.3	0.51	0.39	0.65***
All Africa	554	7.8	87.9	0.77	0.71	0.84***	316	6.5	53	0.66	0.59	0.73***
Middle East												
Iraq	518	7.3	85.7	0.65	0.60	0.71***	342	5.6	55.1	0.63	0.57	0.70***
Syria	201	2.0	94.1	0.69	0.60	0.80***	147	1.9	58.9	0.70	0.59	0.82***
Lebanon	174	2.4	99.4	0.73	0.63	0.85***	91	1.9	50.9	0.52	0.42	0.65***
Iran	434	6.1	63.4	0.48	0.44	0.53***	369	5.2	47.8	0.55	0.50	0.61***
All Middle east	1327	17.8	79.1	0.60	0.57	0.63***	949	14.5	52.3	0.60	0.56	0.64***
Asia												
India	71	1.1	67.7	0.56	0.45	0.71***	58	1.2	57.2	0.63	0.48	0.81***
Vietnam	104	1.1	101.4	0.75	0.62	0.92***	75	1.2	53.6	0.55	0.44	0.69***
Thailand	23	0.5	131.0	1.18	0.77	1.79	121	2.7	50.8	0.62	0.52	0.74***
China	123	0.9	87.7	0.58	0.49	0.70***	123	1.2	64.3	0.65	0.54	0.77***
All Asia	650	8.7	86.3	0.66	0.61	0.71***	738	12.5	61.8	0.65	0.60	0.70***

*Note*: HRs—adjusted for age, education, income, marital status, and calendar years.

*** *p* < 0.001.

** *p* < 0.01.

* *p* < 0.05.

### 3.2 Infection‐associated cancers

#### 3.2.1 Stomach cancer

**FIGURE 2:**
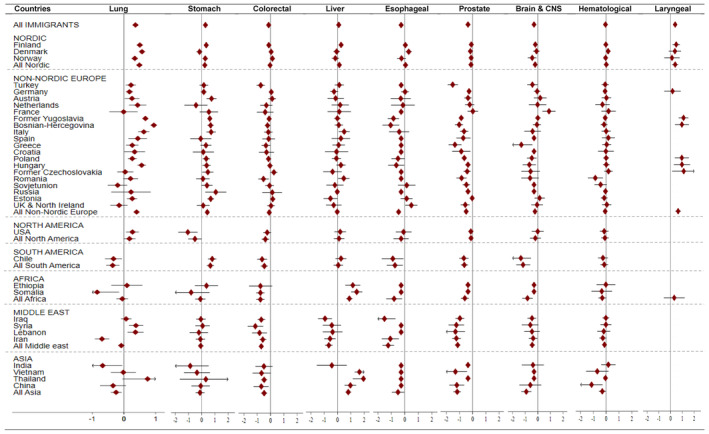
Hazard ratios (HR) (in logarithmic scale) with 95% CI for selected cancer types among *male immigrants*, compared to Sweden‐born individuals, Sweden, 1992 to 2016. The exponentiated log‐HRs are available in Data S1. The HR is adjusted for age, education, income, marital status, and calendar years. HRs not reported for regions/countries with less than 5 reported deaths with respective cancer types.

**FIGURE 3:**
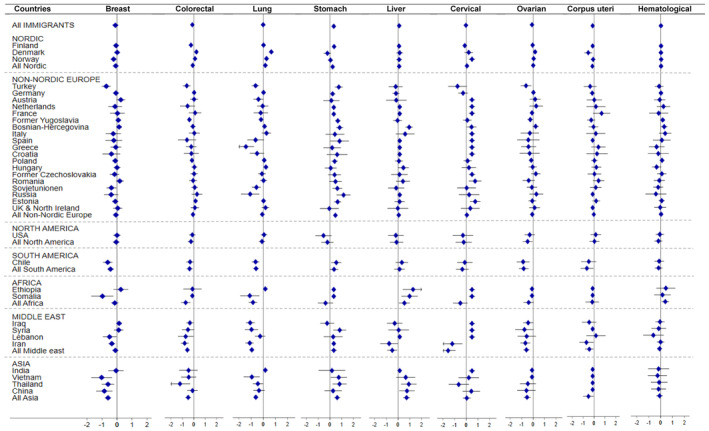
Hazard ratios (HR) (in logarithmic scale) with 95% CI for selected cancer types among *female immigrants*, compared to Sweden‐born individuals, Sweden, 1992 to 2016. The exponentiated log‐HRs are available in Data S1. The HR is adjusted for age, education, income, marital status, and calendar years. HRs not reported for regions/countries with less than 5 reported deaths with respective cancer types.

The mortality rate of stomach cancer was significantly higher among immigrant men and women compared to Swedish‐born individuals (HR_f_ = 1.39: 1.30, 1.49; HR_m_ = 1.33: 1.26, 1.41) (Table 3). At the country of birth level, a significantly elevated risk of stomach cancer morality was observed among male and female immigrants from the Nordic countries particularly from Finland, and some non‐Nordic European countries, mainly countries in Central and South East Europe. The highest risk was observed for Russians (HR_f_ = 3.19: 1.76, 5.79; HR_m_ = 2.91: 1.31, 6.50) followed by Estonians (HR_m_ = 1.98: 1.55, 2.53; HR_f_ = 1.93: 1.44, 2.59). However, we observed a significantly lower mortality rate of stomach cancer in male immigrants from the United States (HR_m_ = 0.35: 0.17, 0.73) (Figures 2 and 3).

**TABLE 3:** Mortality risk by cancer type and gender in migrant groups, compared to Sweden‐born individuals, Sweden, 1992 to 2016.

Cancer type	Female			Male		
	Cancer deaths (Immigrants/Swedish)	HRs (95% CI)		Cancer deaths (Immigrants/Swedish)	HRs (95% CI)	
		Model 1	Model 2		Model 1	Model 2
Infection‐associated cancers						
Stomach	1133/7091	1.40 (1.32, 1.49)***	1.39 (1.31, 1.49)***	1442/10,317	1.41 (1.33, 1.49)***	1.33 (1.26, 1.41)***
Liver	740/5642	1.15 (1.07, 1.25)***	1.10 (1.01, 1.19)**	1105/7908	1.35 (1.27, 1.44)***	1.10 (1.02, 1.17)**
Cervical	489/3213	1.12 (1.02, 1.24)**	0.97 (0.87, 1.07)	N/A	N/A	N/A
Lifestyle‐related cancers						
Lung	4258/30,688	1.08 (1.05, 1.12)***	0.99 (0.96, 1.03)	6670/37,993	1.67 (1.63, 1.71)***	1.44 (1.40, 1.49)***
Esophageal	311/2372	1.14 (1.01, 1.28)*	1.07 (0.94, 1.21)	588/6212	0.87 (0.80, 0.95)**	0.76 (0.69, 0.83)***
Laryngeal	34/196	1.47 (1.02, 2.12)**	1.23 (0.84, 1.81)	180/960	1.84 (1.57, 2.16)***	1.47 (1.24, 1.75)***
Colorectal	2857/28,663	0.89 (0.86, 0.93)***	0.88 (0.85, 0.92)***	2739/29,426	0.94 (0.91, 0.98)***	0.87 (0.83, 0.90)***
Pancreas	1935/17,947	0.93 (0.89, 0.97)**	0.91 (0.87, 0.96)***	1682/15,682	1.02 (0.97, 1.08)	0.93 (0.88, 0.98)**
Hormone‐related cancers						
Breast	3755/32,613	0.91 (0.88, 0.94)***	0.90 (0.87, 093)***	N/A	N/A	N/A
Corpus Uteri	749/7218	0.90 (0.84, 0.97)**	0.88 (0.81, 0.95)**	N/A	N/A	N/A
Ovarian	1543/13,339	0.89 (0.85, 0.94)***	0.91 (0.86, 0.96)**	N/A	N/A	N/A
Prostate	N/A	N/A	N/A	3371/55,795	0.73 (0.71, 0.76)**	0.70 (0.68, 0.73)*
Other cancers						
Brain and CNS	721/6484	0.83 (0.77, 0.90)***	0.82 (0.76, 0.89)***	841/8262	0.83 (0.77, 0.89)***	0.74 (0.68, 0.79)***
Kidney	631/6081	0.90 (0.83, 0.98)**	0.92 (0.85, 1.01)	830/8369	0.96 (0.90, 1.03)	0.92 (0.85, 0.99)**
Bladder	426/4288	0.96 (0.87, 1.06)	0.91 (0.82, 1.01)	905/9998	1.02 (0.95, 1.09)	0.94 (0.87, 1.01)
Hematological^a^	1950/17,330	1.00 (0.96, 1.05)	1.02 (0.97, 1.07)	1956/19,831	0.99 (0.95, 1.04)	0.95 (0.90, 0.99)*
Malignant Melanoma	324/3946	0.64 (0.57, 0.72)***	0.64 (0.57, 0.72)***	392/5608	0.62 (0.56, 0.68)***	0.57 (0.51, 0.63)***
Gall bladder	399/4612	0.74 (0.67, 0.82)***	0.78 (0.70, 0.87)***	150/1868	0.80 (0.68, 0.95)**	0.85 (0.71, 1.01)
Vulva and vagina	157/1834	0.81 (0.69, 0.96)**	0.80 (0.68, 0.95)**	N/A	N/A	N/A
Thyroid	100/1077	0.86 (0.70, 1.06)	0.83 (0.67, 1.02)	82/618	1.29 (1.03, 1.63)**	1.17 (0.91, 1.49)
Mesothelioma	67/347	1.54 (1.19, 2.01)**	1.44 (1.10, 1.90)**	228/1851	1.16 (1.01, 1.33)*	1.06 (0.92, 1.22)
Head and neck^b^	238/2049	0.99 (0.86, 1.13)	0.87 (0.76, 1.00)	462/3656	1.12 (1.02, 1.23)*	0.84 (0.76, 0.94)**

*Note*: Model 1—Adjusted only for age; Model 2—adjusted for age, education, income, marital status, and calendar year.

^a^ Includes leukemia, Hodgkin lymphoma, non‐Hodgkin lymphoma, and multiple myeloma.

^b^ Includes pharyngeal (oropharynx, hypopharynx, and nasopharynx), lip, and oral cancers.

*** *p* < 0.001.

** *p* < 0.01.

* *p* < 0.05.

#### 3.2.2 Liver cancer

We observed a 10% elevated mortality rate of liver cancer both in immigrant men (HR_m_ = 1.10: 1.02, 1.17) and women (HR_m_ = 1.10: 1.01, 1.19) compared to their Swedish‐born counterparts (Table 3), particularly among those from the Nordic (HR_m_ = 1.15: 1.04, 1.27), African (HR_m_ = 2.44: 1.93, 3.07; HR_f_ = 1.71: 1.07, 2.73), and Asian (HR_m_ = 2.25: 1.82, 2.79; HR_f_ = 2.06: 1.55, 2.74) regions (Figures 2 and 3). At the country level, we found significantly higher mortality rates of liver cancer in immigrants from Ethiopia (HR_f_ = 3.56: 1.48, 8.57, HR_m_ = 3.16: 1.79, 5.58), Somalia (HR_f_ = 2.66: 1.32, 5.36; HR_m_ = 4.24: 2.77, 6.49), Thailand (HR_f_ = 2.49: 1.29, 4.81; HR_m_ = 7.04: 3.15, 15.71), China (HR_f_ = 2.13: 1.11, 4.11; HR_m_ = 2.63: 1.69, 4.08), among female immigrants from Bosnia‐Herzegovina (HR_f_ = 2.57: 1.89, 3.49), and male immigrants from Vietnam (HR_m_ = 5.20: 3.62, 7.46), Italy (HR_m_ = 1.65: 1.09, 2.51), and Romania (HR_m_ = 1.60: 1.04, 2.46). However, the risk was found significantly lower among males from the Middle East (HR_m_ = 0.52: 0.39, 0.69) (Figures 2 and 3).

#### 3.2.3 Cervical cancer

The mortality rate of cervical cancer was not significantly different between immigrant and non‐immigrant women (HR = 0.97: 0.87, 1.07) (Table 3). However, immigrants from certain countries including Norway (HR = 1.63: 1.25, 2.13), Bosnia‐Herzegovina (HR = 1.57: 1.03, 2.40), Romania (HR = 2.13: 1.23, 3.68), and Estonia (HR = 2.15: 1.37, 3.38) exhibited a higher mortality rate. Conversely, immigrants from the Middle East (HR = 0.21: 0.11, 0.40) particularly from Iran (HR = 0.30: 0.12, 0.72) experienced a significantly lower risk of cervical cancer death in comparison with women born in Sweden (Figure 3).

### 3.3 Tobacco‐related cancers

#### 3.3.1 Lung cancer

A significantly increased mortality rate of lung cancer was observed in male immigrants (HR_m_ = 1.44: 1.40, 1.49) in comparison with the Swedish‐born population (Table 3). There was considerable variation by country of birth. Immigrants from the Nordic countries exhibited a significantly higher risk in both genders (HR_f_ = 1.18: 1.14, 1.24; HR_m_ = 1.63: 1.57, 1.69). For the majority of non‐Nordic European countries, the elevated risk was observed only among male immigrants, with the highest risk for immigrants from Bosnia (HR_m_ = 2.57: 2.32, 2.85) followed by the former Yugoslavia (HR_m_ = 1.97: 1.81, 2.13). Among females, we observed a lower mortality rate among immigrants from Africa (HR_f_ = 0.43: 0.30, 0.61), Middle East (HR_f_ = 0.39: 0.32, 0.47), Asia (HR_f_ = 0.54: 0.44, 0.66), and a few non‐Nordic European countries including Turkey (HR_f_ = 0.53: 0.39, 0.71), Greece (HR_f_ = 0.24: 0.12, 0.47), Russia (HR_f_ = 0.34: 0.16, 0.72), and the former Soviet Union (HR_f_ = 0.57: 0.39, 0.81) compared to individuals born in Sweden. Immigrants from South America (HR_f_ = 0.55: 0.43, 0.70; HR_m_ = 0.70: 0.57, 0.87) also showed a lower risk for lung cancer mortality (Figures 2 and 3).

#### 3.3.2 Esophageal cancer

Compared to Swedish‐born individuals, the mortality rate of esophageal cancer was significantly lowered among male immigrants (HR_m_ = 0.76: 0.69, 0.83) (Table 3). However, the risk of death due to this cancer was higher in female immigrants from Finland (HR_f_ = 1.23: 1.02, 1.49), Somalia (HR_f_ = 5.18: 2.29, 11.72), as well as male immigrants from Denmark (HR_m_ = 1.33: 1.06, 1.67), and the UK and Northern Ireland (HR_m_ = 1.62: 1.03, 2.54) compared to individuals born in Sweden. Conversely, male immigrants from Africa (HR_m_ = 0.45: 0.25, 0.82), Middle East (HR_m_ = 0.29: 0.19, 0.45), and some non‐Nordic European countries including Yugoslavia (HR_m_ = 0.43: 0.28, 0.65), Bosnia‐Hercegovina (HR_m_ = 0.35: 0.19, 0.66), Poland (HR_m_ = 0.60: 0.37, 0.98), Hungary (HR_m_ = 0.53: 0.29, 0.96), and those from South America (HR_m_ = 0.48: 0.26, 0.86) demonstrated significantly lower mortality rate compared to the local born individuals in Sweden (Figure 2; Table S2).

#### 3.3.3 Laryngeal cancer

We observed about 50% significantly higher mortality rate of laryngeal cancer in male immigrants (HR_m_ = 1.47: 1.24, 1.75) (Table 3). Notably, male immigrants from Finland (HR_m_ = 1.61: 1.18, 2.19), former Yugoslavia (HR_m_ = 2.99: 1.98, 4.52), Bosnia‐Herzegovina (HR_m_ = 2.61: 1.44, 4.75), Poland (HR_m_ = 2.59: 1.42, 4.72), Hungary (HR_m_ = 2.62: 1.31, 5.27), and former Czechoslovakia (HR_m_ = 3.05: 1.14, 8.15), as well as female immigrants from Norway (HR_f_ = 2.46: 1.09, 5.57) showed higher mortality rates of laryngeal cancer compared to non‐immigrants (Figure 2; Table S2).

#### 3.3.4 Colorectal cancer

With few exceptions including female immigrants from Denmark (HR_f_ = 1.25: 1.11, 1.42) and Norway (HR_f_ = 1.11: 1.00, 1.23), and male immigrants from Norway (HR_m_ = 1.15: 1.01, 1.31) who exhibited a significantly higher risk of mortality (Figures 2 and 3), overall, immigrant women and men had a lower mortality rate of colorectal cancer (HR_f_ = 0.88: 0.85, 0.92; HR_m_ = 0.87: 0.83, 0.90) compared with the Swedish‐born individuals (Table 3).

### 3.4 Head and neck cancers

The risk of mortality stemming from lip, oral cavity, and pharyngeal cancers combined was significantly lower among male immigrants (HR_m_ = 0.84: 0.76, 0.94) than Swedes (Table 3), whereas there was significantly higher risk of death among immigrants from specific countries/regions including Finland (HR_m_ = 1.21: 1.02, 1.43), India (HR_m_ = 2.55: 1.21, 5.36), France (HR_m_ = 2.76: 1.31, 5.80), former Czechoslovakia (HR_m_ = 1.90: 1.05, 3.44), and Estonia (HR = 1.78: 1.12, 2.83). Among female immigrants, the risk of mortality from these head and neck cancers was inconclusive, although those from Somalia (HR_f_ = 5.40: 2.52, 11.55) and Austria (HR_f_ = 2.67: 1.20, 5.95) showed a significantly higher risk (Table S2).

### 3.5 Hormone‐related cancers

#### 3.5.1 Breast cancer

Overall immigrants showed a 10% significantly lower risk of female breast cancer mortality compared to Swedish‐born women (HR = 0.90: 0.87, 0.93) (Table 3), ranging from 8% in the Nordic countries (HR = 0.92: 0.88, 0.97) to about 60% among immigrants from Vietnam (HR = 0.36: 0.18, 0.72). (Figure 3). However, the majority of non‐Nordic European immigrants did not display a difference compared to Swedes (Figure 3).

#### 3.5.2 Corpus‐uteri and ovarian

The risk of mortality for corpus‐uteri and ovarian cancers was significantly lower in immigrants (HR = 0.88: 0.81, 0.95; HR = 0.91: 0.86, 0.96, respectively) than women born in Sweden (Table 3), although no difference was observed for most immigrants at country‐specific level. The lowest rate was observed among Iranian women for corpus‐uteri cancer (HR = 0.50: 0.26, 0.96) and Chilean women (HR = 0.42: 0.24, 0.74) for ovarian cancer (Figure 3).

#### 3.5.3 Prostate cancer

Immigrant men exhibited a 30% lower risk for prostate cancer mortality (HR = 0.70: 0.68, 0.73) in comparison with men born in Sweden. This lower mortality rate in the immigrant group appeared consistent for most country‐specific estimates, ranging from a 10% lower risk in immigrants from Finland (HR = 0.90: 0.84, 0.96) to a substantial 78% lower risk among Turkish men (HR = 0.22: 0.15, 0.33) (Figure 2).

### 3.6 Other cancers

#### 3.6.1 Brain cancer

The risk of brain and CNS cancer mortality was significantly lower among immigrant women (HR_f_ = 0.82: 0.76, 0.89) and men (HR_m_ = 0.74: 0.68, 0.79) compared to non‐immigrants (Table 3). The results were also consistent when analyzed at region or country levels, the risk being lowest among immigrants from South America (HR_f_ = 0.33: 0.17, 0.63 and HR_m_ = 0.31: 0.18, 0.55). However, male immigrants from France exhibited a significantly higher risk of death (HR_m_ = 2.43: 1.44, 4.10) compared to the Swedish‐born individuals (Figure 2; Table S2).

#### 3.6.2 Malignant melanoma

Compared to the Swedish‐born people, overall immigrants had a significantly lower risk of mortality due to malignant melanoma (HR_f_ = 0.64: 0.57, 0.72; HR_m_ = 0.57: 0.51, 0.63) (Table 3). The lowest risk of mortality was observed among immigrants from the Middle East (HR_f_ = 0.13: 0.06, 0.32 and HR_m_ = 0.16: 0.09, 0.29) (Table S1).

#### 3.6.3 Mesothelioma cancer

Compared to the non‐immigrants, the mortality rate of mesothelioma cancer was significantly higher in female immigrants (HR_f_ = 1.44: 1.10, 1.90) (Table 3). At the region or country level, male immigrants from the Nordic (HR_m_ = 1.26: 1.03, 1.53) and female immigrants from Denmark (HR_m_ = 2.96: 1.47, 5.98) exhibited a significantly higher mortality rate. Turkish men (HR_f_ = 11.92: 6.16, 23.06) and women (HR_m_ = 2.34: 1.35, 4.06) had an exceptionally higher risk of death for mesothelioma cancer (Tables S1 and S2).

#### 3.6.4 Hematological cancer

There were no significant differences on mortality rate of hematological malignancies among female immigrants compared to Sweden‐born women (Table 3), except for immigrants from Bosnian (HR = 134: 1.02, 1.76), and those from Africa (HR = 1.47: 1.07, 2.02) who exhibited a significantly higher mortality rate. In men, we found a 5% lower mortality rate in all immigrants compared to the Swedish‐born population (HR_m_ = 0.95: 0.90, 0.99), and among Asian immigrants (HR_m_ = 0.74: 0.57, 0.97) at the region level. An exception was Danish male immigrants who had a significantly higher mortality rate (HR_m_ = 1.17: 1.02, 1.35) than non‐immigrants (Figures 2 and 3).

## 4 DISCUSSION

This study investigated disparities in cancer mortality by country of birth and cancer type in Sweden while also considering potential gender differences. The study highlights that immigrants generally experience a somewhat lower overall cancer mortality rate compared to Swedish‐born. This finding aligns with previous research, both from Sweden^6^, ^8^, ^9^ and internationally,^23^, ^24^, ^25^ which has consistently suggested that immigrants experience a reduced risk of all‐cause cancer‐related mortality. This is also in line with the “healthy immigrant paradox” suggesting that immigrants constitute a selection of younger and healthier individuals from their country of origin.^26^ By performing a more detailed analysis of specific cancer types by country of birth, we were able to show an unambiguous and more complex pattern. Some immigrant groups face an increased risk of mortality from specific cancers, most notably lung, liver, laryngeal, and stomach cancers; and the risk was lower for colorectal, breast, and prostate cancers in most immigrant groups, when compared to non‐immigrants. Prior studies conducted in Sweden^8^, ^9^ and also in other European countries, Australia, and the United States support these findings.^27^, ^28^, ^29^, ^30^

The etiology and risk factors vary greatly by cancer type. For infection‐related cancers like liver, stomach, and cervical cancer, pre‐migration factors play a pivotal role in explaining the disparities in cancer mortality between immigrants and non‐immigrants.^31^ Many immigrant groups encounter barriers to healthcare access in their home countries (such as vaccine availability or prevention of specific infections), which can result in delayed detection and treatment of infections that contribute to these cancers. This is particularly evident in the context of chronic hepatitis B virus (HBV) infections, which is the main risk factor for liver cancer in many regions of the world. HBV infection is more prevalent among immigrants due to lower vaccination and treatment rates in their countries of origin.^32^ In 2016, the European Centre for Disease Prevention and Control (ECDC) report showed that immigrants account for an estimated 25% of the HBV cases in the EU/EEA.^33^ A previous study in Sweden also shows that a significant proportion of hepatitis B carriers are immigrants from non‐Western countries, including Asian and African nations.^34^ Chronic HBV infections are relatively rare among native Swedes,^35^ and this contrast in the prevalence of HBV infections could explain the observed disparities in liver cancer mortality. Furthermore, due to limited healthcare availability and standards in many immigrant nations, chronic liver diseases like HBV infection can progress asymptomatically over an extended period.^36^ This can lead to advanced‐stage liver cancer at the time of diagnosis which cannot be treated anymore.

Similarly, the prevalence of *Helicobacter pylori (H. pylori*) infection, a well‐known risk factor for stomach cancer, is notably high (ranges from 60% to 90%) in low‐resource countries,^37^ and it persists at significant rates in some European regions, particularly Eastern and Southeastern Europe despite improvements in food preservation and storage.^38^ This high infection rate may contribute to the observed disparities in stomach cancer mortality between non‐Nordic European immigrant groups and non‐immigrants in Sweden. Furthermore, the age‐standardized incidence and mortality rates of stomach cancer are higher in central‐east and southern Europe than in the northern European countries.^39^ The delayed detection and treatment of *H. pylori* infections, combined with other lifestyle factors like diet, smoking, and excessive alcohol consumption,^39^ may further exacerbate the risk of stomach cancer mortality among immigrants. However, some findings may require further investigation. For example, despite high *H. pylori* infection rates in Africa, the mortality rate of stomach cancer appears lower among African immigrants in Sweden. This phenomenon, often referred to as the “African enigma”,^40^ suggests that factors beyond *H. pylori* infection may be at play in this specific case. Additionally, the higher mortality rate of stomach cancer in the Nordic regions may not be explained by *H. pylori* infection, as low infection rates are reported in these countries. Lifestyle factors, such as smoking, as well as body weight which is associated with cardia and stomach cancer may explain this difference.

Estimating the mortality of laryngeal cancer was limited to few immigrant groups due to rarity of the disease. However, the available data suggested that certain immigrant groups, particularly male immigrants from Nordic and specific European countries, exhibited significantly higher mortality risk compared to non‐immigrants. Likewise, a heightened risk of lung cancer mortality was observed among similar immigrant groups from Nordic and several European countries, as well as male immigrants from Middle Eastern countries. The results are consistent with prior research conducted in Sweden.^8^ The primary factors contributing to the increased mortality rates of both laryngeal and lung cancer appear to be cigarette smoking, which accounts for about 90% of worldwide mortality for laryngeal,^41^ and more than 80% in the United States and France and 40% in sub‐Sahara Africa for lung cancer.^42^ The higher prevalence of smoking in several non‐Nordic European countries is possibly influenced by historical factors such as wartime and post‐war periods, along with other lifestyle factors, and may explain the elevated mortality risk of this cancer among European immigrant groups compared to non‐immigrants in Sweden where the prevalence of cigarette smoking is notably lower than most European countries.^43^

The study also revealed that certain immigrant groups, such as those from Africa, exhibited notably higher mortality rate of esophageal and head and neck cancers, compared to individuals born in Sweden. Incidence of esophageal and pharyngeal cancers has previously been reported as significantly higher among immigrant groups in Sweden.^44^ Given that these types of cancers are predominately associated with smoking,^45^ the discrepancy might be attributed, in part, to the relatively low prevalence of tobacco smoking within the Swedish population. Moreover, early diagnosis and treatment can significantly impact the outcomes (progression and metastasis) of esophageal and head and neck cancers, and thus, differences in healthcare access before migration appear to play a crucial role and could contribute to disparities in mortality rates between immigrant groups and Swedish‐born individuals. Lifestyle factors like alcohol consumption and unhealthy diet, infections (HBV and Epstein Barr virus), and low socioeconomic status could also further contribute to explaining these disparities.

The risk of mesothelioma mortality also exhibited a notably elevated risk among immigrants, particularly those originating from the Nordic countries and Turkey. Mesothelioma is a relatively rare cancer, primarily caused by asbestos exposure.^46^ To the best of our knowledge, no previous report is available on the incidence or mortality rates of Mesothelioma among immigrants in European countries. Some studies conducted in Sweden in the 1980s and studied the risk of Mesothelioma in immigrant cohorts with a history of exposure to occupational hazards in their home country. These studies reported a higher incidence of mesothelioma cancer among Turkish^9^, ^47^ and Danish^9^ immigrant men in Sweden. A recent study using the Global Cancer Observatory database revealed that the highest incidence rate of mesothelioma cancer was observed in North Europe, possibly due to better diagnosis and more complete registration.^48^ Sweden banned the use of asbestos in new construction projects in the early 1980s; however, given its long latency period, often ranging from 20 to 50 years between asbestos exposure and the development of the disease, immigrants who moved to Sweden in the aftermath of World War II to work in industries such as construction, shipbuilding, mining, and insulation manufacturing may have been exposed to asbestos. This fact complemented by their pre‐migration history of asbestos exposure might be the reason for excess mortality risk among these groups. It is, however, important to approach these findings with caution due to the relatively low number of reported deaths.

With some exceptions, such as the increased risk of cervical, pancreas, and colorectal cancers among some immigrant groups, immigrants typically experience a lower mortality rate for most other cancer types including breast, prostate, hematological, gynecological, melanoma, gall bladder, kidney, and thyroid cancers. This pattern has been confirmed in many other studies for breast, prostate,^8^, ^9^ and gynecological^9^ cancers. As immigrants often maintain their cultural practices, dietary habits, and lifestyle choices with them when they move to a new country, these can influence the risk of mortality from these cancers. The “healthy migrant effect” may also further contribute to this trend. For example, compared to dietary habits in Western countries like Sweden, diets in Africa and Asia are characterized by their reduced consumption of processed foods and higher intake of fiber.^49^ This dietary contrast may be a contributing factor to the observed lower rates of colorectal cancer mortality among immigrants from these regions. Genetic factors, as some immigrant groups may have genetic predispositions that are associated with lower cancer incidence and mortality,^50^ might contribute further to the observed lower risk of mortality, for example, in the case of breast and cervical cancers. However, the observed lower cancer mortality rates among immigrants are not a universal trend and can depend on a multitude of factors including adapting to a new way of life in the host country—the phenomenon called acculturation, and thus, in the long run, mortality advantage for certain cancer types may diminish. Therefore, an ongoing monitoring of this population segment regarding mortality risk disparities by cancer types is essential.

These findings will be instrumental in enhancing our understanding of cancer epidemiology among immigrants in Sweden and developing targeted interventions and policies that aim at reducing the cancer burden in this segment of the population within the national cancer control program. Given that the observed mortality rates may be closely tied to the incidence of some cancers for immigrants in their home countries, it is also imperative to boost cancer screening initiatives and tobacco control measures among immigrants. Establishing a comprehensive surveillance system to enhance screening and early detection of cancer in newcomers would be a necessary step to consider. Moreover, the changing demographics in composition and aging migrant population in Sweden—in which cancer incidence and mortality are prevalent, also necessitate ongoing monitoring, and further studies to explore cancer mortality disparities by behavioral and socio‐cultural and economic risk factors, considering age and calendar period effects, as well as the economic implications within the immigrant population are warranted.

## 5 STRENGTH AND LIMITATIONS

The study used high‐quality total population data with a long‐term follow‐up and provided comprehensive evidence on cancer mortality rates among distinct groups of immigrants in Sweden on various site‐specific cancer types. Another strength is that the estimates that were adjusted for socio‐demographic factors. Income and education status were strong contributing factors for all‐cause mortality disparities between immigrants and Swedish‐born individuals in a previous Swedish study.^7^ There are also limitations in this study that need to be considered. First, immigrants returning to their country of birth when they become seriously ill might cause bias and leading to underestimates of cancer mortality in immigrant groups, a phenomenon called salmon bias. Nevertheless, it is essential to consider opposing viewpoints also, as some scholars argue that since access to and quality of cancer health care in many immigrants' home nations are not advanced, immigrants may be less inclined to move back to their home countries. This is evidenced in a Danish study among immigrants, which showed a higher disease severity was associated with fewer emigrations.^51^ In our study, loss to follow‐up due to emigration was highest among immigrants from France (~44%), while the lowest was from Syria (~3%) followed by Eritrea (~4%). For the majority of immigrant groups, loss to follow‐up due to emigration was between 10% and 20%. Second, exclusively socio‐demographic factors are used as confounders. However, it is important to note that estimates on cancer mortality rates are also determined by other factors such as health behaviors (e.g., smoking, diet, alcohol, and physical activity), medical‐related factors, genetic risk factors, and environmental hazards. Thus, a comprehensive analysis that takes into account behavioral, cultural, socioeconomic, and biological factors, as well as the impact of acculturation or post‐migration lifestyle changes, and barriers to healthcare access could potentially offer a relatively better understanding of cancer disparities in the immigrant population in Sweden. In addition, the study exclusively relied on cancer mortality data due to data limitations. Cancer mortality data alone might not provide a comprehensive understanding of cancer disparities. It is essential to consider factors such as incidence and survival rates—for example, reporting mortality‐to‐incidence rate rations.

## 6 CONCLUSIONS

This study reveals that while immigrant groups exhibited somewhat lower mortality rates for overall and some specific cancer types, certain groups face a higher risk of mortality, particularly for cancers linked to infections and tobacco use, as compared with non‐immigrants. Policies and prevention actions should especially focus on cancers related to infections and tobacco to reduce cancer mortality in immigrants.

## AUTHOR CONTRIBUTIONS

**Daniel Nigusse Tollosa:** Conceptualization (supporting); data curation (lead); formal analysis (lead); methodology (equal); visualization (lead); writing – original draft (lead). **Kazem Zendehdel:** Conceptualization (equal); data curation (supporting); formal analysis (supporting); investigation (equal); methodology (supporting); supervision (equal); visualization (supporting); writing – review and editing (equal). **Alessandro Procopio:** Formal analysis (supporting); methodology (supporting); writing – review and editing (equal). **Agneta Cederström:** Formal analysis (supporting); methodology (supporting); writing – review and editing (equal). **Paolo Boffetta:** Conceptualization (equal); writing – review and editing (equal). **Eero Pukkala:** Formal analysis (supporting); methodology (supporting); writing – review and editing (equal). **Mikael Rostila:** Conceptualization (equal); formal analysis (supporting); funding acquisition (lead); methodology (equal); project administration (supporting); resources (lead); supervision (lead); writing – review and editing (equal).

## CONFLICT OF INTEREST STATEMENT

All authors declared no conflict of interest.

## Supporting information


Data S1.

## Data Availability

The findings of this study are supported by data accessible through The Swedish Public Health Agency. However, restrictions are in place due to the sensitive nature of individual‐level health data. Following ethical approval from the ethical review agency is necessary to address the research question, a data request can be submitted to the respective authority. that is, registerhantering@folkhalsomyndigheten.se. Data may be obtainable from the authors upon reasonable request and with permission from The Swedish Public Health Agency.
