# Supplementary material for: Cancer mortality by country of birth and cancer type in Sweden: A 25‐year registry‐based cohort study
Source: Cancer Med. 2024 Jul 17;13(14):e70020. doi: 10.1002/cam4.70020 (PMC11253184; doi:10.1002/cam4.70020)
Supplement: Supplementary file 1 — Data S1. [file CAM4-13-e70020-s001.zip › cam470020-sup-0001-Figures.docx]

**Supplementary figures (sFigure 1 and 2)**

**(A)**

**(B)**

**sFigure 1** - all-site cancer mortality rates (age standardized) by calendar periods among immigrant groups and Sweden-born, males **(A)** and females **(B)**. Non-western immigrants include those from South America, Asia, Africa, and the Middle-East

**(A)**

**(B)**

**sFigure 2** - All-site cancer mortality rates (age standardized) by age groups among immigrant groups and Sweden-born, males **(A)** and females **(B)**. Non-western immigrants include those from South America, Asia, Africa, and the Middle-East
