# Supplementary material for: Cancer mortality by country of birth and cancer type in Sweden: A 25‐year registry‐based cohort study
Source: Cancer Med. 2024 Jul 17;13(14):e70020. doi: 10.1002/cam4.70020 (PMC11253184; doi:10.1002/cam4.70020)
Supplement: Supplementary file 1 — Data S1. [file CAM4-13-e70020-s001.zip › cam470020-sup-0002-Tables.docx]

**Supplementary materials**

sTable 1 – Person years and mortality risk estimates (Crude and Age standardized mortality rates, and Hazard ratio (HRs) with 95% CI) by cancer types and immigrants’ groups, **males,** 1992 to 2016, Sweden

| Countries/region | **All cancers** | | | | | | | **Stomach** | | | | | | | **Liver** | | | | | | **Lung** | | | | | |
| --- | --- | --- | --- | --- | --- | --- | --- | --- | --- | --- | --- | --- | --- | --- | --- | --- | --- | --- | --- | --- | --- | --- | --- | --- | --- | --- |
|  | Person years in 100,000 | Total deaths | CMR per 100,000 | ASMR per 100,000 | Hazard ratio, 95% CI | | | Total deaths | CMR per 100,000 | ASMR per 100,000 | Hazard ratio, 95% CI | | | Total deaths | | CMR per 100,000 | ASMR per 100,000 | Hazard ratio, 95% CI | | | Total deaths | CMR per 100,000 | ASMR per 100,000 | Hazard ratio, 95% CI | | |
| **SWEDEN (ref)** | **717.6** | **251,509** | 350.5 | 106.4 | 1.00 |  |  | **10,317** | 14.4 | 4.3 | 1.00 |  |  | **7,908** | | 11.0 | 3.5 | 1.00 |  |  | **37,993** | 52.9 | 17.3 | 1.00 |  |  |
| **IMMIGRANTS** | **106.4** | **26,178** | 246.0 | 113.7 | 0.97 | 0.95 | 0.98 | **1,442** | 13.6 | 6.3 | 1.33 | 1.26 | 1.41 | **1,105** | | 10.4 | 4.9 | 1.10 | 1.02 | 1.17 | **6,670** | 62.7 | 29.3 | 1.44 | 1.40 | 1.49 |
| Finland | **16.3** | **6,696** | 411.5 | 123.6 | 1.11 | 1.09 | 1.14 | **336** | 20.6 | 6.3 | 1.41 | 1.26 | 1.57 | **284** | | 17.5 | 5.1 | 1.31 | 1.16 | 1.48 | **1,808** | 111.1 | 31.8 | 1.65 | 1.57 | 1.73 |
| Denmark | **4.2** | **2,587** | 613.0 | 135.1 | 1.15 | 1.10 | 1.19 | **76** | 18.0 | 4.0 | 0.85 | 0.67 | 1.06 | **78** | | 18.5 | 4.1 | 0.94 | 0.74 | 1.19 | **678** | 160.7 | 36.6 | 1.77 | 1.63 | 1.91 |
| Norway | **3.4** | **1,921** | 557.5 | 124.3 | 1.04 | 0.99 | 1.09 | **97** | 28.2 | 6.2 | 1.31 | 1.07 | 1.61 | **52** | | 15.1 | 4.2 | 0.85 | 0.65 | 1.12 | **415** | 120.4 | 27.7 | 1.41 | 1.27 | 1.55 |
| **All NORDIC** | **24.2** | **11,247** | 464.4 | 125.9 | 1.11 | 1.09 | 1.13 | **512** | 21.1 | 5.8 | 1.27 | 1.16 | 1.39 | **416** | | 17.2 | 4.7 | 1.15 | 1.04 | 1.27 | **2,913** | 120.3 | 32.2 | 1.63 | 1.57 | 1.69 |
| Turkey | **4.0** | **509** | 127.2 | 94.1 | 0.70 | 0.64 | 0.76 | **39** | 9.7 | 6.9 | 1.18 | 0.85 | 1.64 | **33** | | 8.2 | 5.8 | 1.14 | 0.81 | 1.62 | **158** | 39.5 | 30.2 | 1.25 | 1.06 | 1.46 |
| Germany | **3.8** | **1,789** | 469.6 | 108.1 | 0.98 | 0.93 | 1.02 | **84** | 22.1 | 5.4 | 1.17 | 0.93 | 1.46 | **49** | | 12.9 | 3.0 | 0.77 | 0.58 | 1.04 | **344** | 90.3 | 20.9 | 1.19 | 1.07 | 1.33 |
| Austria | **0.7** | **358** | 483.8 | 106.6 | 0.99 | 0.89 | 1.09 | **28** | 37.8 | 8.8 | 2.11 | 1.45 | 3.08 | **11** | | 14.9 | 3.0 | 0.87 | 0.47 | 1.62 | **74** | 100.0 | 24.6 | 1.29 | 1.02 | 1.63 |
| Netherlands | **0.6** | **218** | 352.0 | 121.0 | 1.02 | 0.89 | 1.16 | **5** | 8.1 | 3.0 | 0.65 | 0.27 | 1.55 | **9** | | 14.5 | 4.1 | 1.22 | 0.64 | 2.35 | **56** | 90.4 | 31.3 | 1.54 | 1.17 | 2.03 |
| France | **0.6** | **127** | 201.4 | 111.9 | 0.97 | 0.82 | 1.16 | **8** | 12.7 | 7.1 | 1.73 | 0.86 | 3.46 | **5** | | 7.9 | 4.5 | 1.14 | 0.47 | 2.73 | **20** | 31.7 | 19.0 | 0.99 | 0.63 | 1.54 |
| For. Yugoslavia | **7.1** | **1,772** | 251.1 | 124.6 | 1.06 | 1.01 | 1.11 | **124** | 17.6 | 8.5 | 1.85 | 1.54 | 2.21 | **65** | | 9.2 | 4.4 | 0.99 | 0.78 | 1.27 | **616** | 87.3 | 41.9 | 1.97 | 1.81 | 2.13 |
| Bosnian-Hercegovina | **4.8** | **990** | 206.3 | 142.7 | 1.13 | 1.06 | 1.21 | **73** | 15.2 | 10.3 | 1.97 | 1.55 | 2.49 | **42** | | 8.8 | 6.6 | 1.10 | 0.81 | 1.51 | **404** | 84.2 | 58.6 | 2.57 | 2.32 | 2.85 |
| Italy | **0.9** | **423** | 452.2 | 124.6 | 1.08 | 0.98 | 1.19 | **31** | 33.1 | 8.8 | 2.05 | 1.43 | 2.93 | **22** | | 23.5 | 6.2 | 1.65 | 1.09 | 2.51 | **126** | 134.7 | 39.0 | 1.87 | 1.56 | 2.23 |
| Spain | **0.6** | **155** | 239.6 | 104.6 | 0.92 | 0.78 | 1.07 | **6** | 9.3 | 3.7 | 0.93 | 0.42 | 2.07 | **8** | | 12.4 | 5.2 | 1.29 | 0.64 | 2.58 | **46** | 71.1 | 29.7 | 1.55 | 1.15 | 2.07 |
| Greece | **1.4** | **316** | 231.4 | 95.5 | 0.76 | 0.68 | 0.85 | **23** | 16.8 | 6.9 | 1.38 | 0.91 | 2.08 | **18** | | 13.2 | 5.1 | 1.09 | 0.68 | 1.75 | **100** | 73.2 | 28.7 | 1.30 | 1.07 | 1.58 |
| Croatia | **0.6** | **107** | 181.7 | 92.6 | 0.78 | 0.65 | 0.95 | **6** | 10.2 | 4.3 | 1.13 | 0.51 | 2.53 | **5** | | 8.5 | 4.4 | 0.93 | 0.39 | 2.24 | **36** | 61.1 | 27.0 | 1.40 | 1.01 | 1.95 |
| Poland | **3.9** | **873** | 226.0 | 109.3 | 0.88 | 0.82 | 0.94 | **56** | 14.5 | 7.6 | 1.41 | 1.08 | 1.85 | **33** | | 8.5 | 4.6 | 0.92 | 0.65 | 1.31 | **201** | 52.0 | 26.9 | 1.30 | 1.13 | 1.50 |
| Hungary | **1.6** | **805** | 490.5 | 122.5 | 1.05 | 0.98 | 1.13 | **42** | 25.6 | 6.0 | 1.45 | 1.06 | 1.97 | **35** | | 21.3 | 6.7 | 1.29 | 0.92 | 1.82 | **224** | 136.5 | 34.0 | 1.75 | 1.53 | 2.00 |
| For. Czechoslovakia | **0.7** | **363** | 511.8 | 115.8 | 0.93 | 0.84 | 1.04 | **24** | 33.8 | 6.3 | 1.60 | 1.05 | 2.43 | **9** | | 12.7 | 2.9 | 0.69 | 0.34 | 1.38 | **63** | 88.8 | 20.9 | 1.04 | 0.81 | 1.35 |
| Romania | **1.4** | **263** | 193.8 | 101.2 | 0.75 | 0.66 | 0.85 | **15** | 11.1 | 6.2 | 1.10 | 0.66 | 1.83 | **21** | | 15.5 | 7.9 | 1.60 | 1.04 | 2.46 | **73** | 53.8 | 28.8 | 1.23 | 0.98 | 1.56 |
| For. Sovjetunionen | **0.6** | **300** | 539.3 | 106.2 | 0.82 | 0.72 | 0.92 | **24** | 43.1 | 10.1 | 1.49 | 0.97 | 2.29 | **10** | | 18.0 | 4.3 | 0.82 | 0.43 | 1.58 | **45** | 80.9 | 16.8 | 0.82 | 0.60 | 1.11 |
| Russia | **0.4** | **42** | 93.6 | 102.1 | 0.82 | 0.61 | 1.11 | **6** | 13.4 | 13.5 | 2.91 | 1.31 | 6.50 | **0** | |  |  |  |  |  | **10** | 22.3 | 31.9 | 1.25 | 0.67 | 2.33 |
| Estonia | **1.0** | **982** | 1007.0 | 121.0 | 1.13 | 1.06 | 1.21 | **67** | 68.7 | 6.6 | 1.98 | 1.55 | 2.53 | **16** | | 16.4 | 1.8 | 0.59 | 0.35 | 1.00 | **163** | 167.1 | 20.9 | 1.30 | 1.10 | 1.52 |
| UK & North Ireland | **2.0** | **313** | 156.3 | 97.1 | 0.82 | 0.73 | 0.91 | **17** | 8.5 | 5.2 | 1.12 | 0.69 | 1.83 | **11** | | 5.5 | 2.9 | 0.75 | 0.42 | 1.36 | **54** | 27.0 | 16.4 | 0.86 | 0.65 | 1.12 |
| **All Non-NORDIC EUROPÉ** | **40.0** | **11,282** | 281.7 | 114.4 | 0.96 | 0.94 | 0.98 | **715** | 17.9 | 7.4 | 1.53 | 1.42 | 1.66 | **418** | | 10.4 | 4.3 | 0.98 | 0.88 | 1.08 | **2,932** | 73.2 | 30.6 | 1.49 | 1.43 | 1.55 |
| USA | **1.4** | **485** | 353.1 | 107.0 | 0.94 | 0.86 | 1.03 | **8** | 5.8 | 2.2 | 0.35 | 0.17 | 0.73 | **21** | | 15.3 | 6.0 | 1.22 | 0.79 | 1.90 | **94** | 68.4 | 23.7 | 1.31 | 1.07 | 1.60 |
| **All North AMERICA** | **2.4** | **590** | 244.3 | 101.4 | 0.91 | 0.84 | 0.98 | **16** | 6.6 | 3.2 | 0.60 | 0.36 | 1.00 | **25** | | 10.4 | 4.9 | 1.12 | 0.75 | 1.68 | **113** | 46.8 | 22.0 | 1.19 | 0.99 | 1.44 |
| Chile | **2.8** | **312** | 112.1 | 94.9 | 0.70 | 0.63 | 0.79 | **40** | 14.4 | 11.3 | 2.22 | 1.61 | 3.06 | **23** | | 8.3 | 7.2 | 1.30 | 0.86 | 1.96 | **59** | 21.2 | 17.8 | 0.72 | 0.55 | 0.94 |
| **All South AMERICA** | **5.0** | **501** | 100.2 | 92.0 | 0.70 | 0.64 | 0.77 | **54** | 10.8 | 9.3 | 1.93 | 1.47 | 2.54 | **30** | | 6.0 | 5.6 | 1.06 | 0.74 | 1.52 | **90** | 18.0 | 16.7 | 0.70 | 0.57 | 0.87 |
| Ethiopia | **1.2** | **74** | 60.8 | 94.7 | 0.84 | 0.67 | 1.05 | **5** | 4.1 | 4.1 | 1.44 | 0.60 | 3.46 | **12** | | 9.9 | 13.2 | 3.16 | 1.79 | 5.58 | **16** | 13.1 | 16.5 | 1.10 | 0.67 | 1.79 |
| Somalia | **1.8** | **65** | 36.0 | 69.5 | 0.60 | 0.47 | 0.76 | **2** | 1.1 | 1.9 |  |  |  | **22** | | 12.2 | 23.9 | 4.24 | 2.77 | 6.49 | **8** | 4.4 | 9.0 | 0.43 | 0.22 | 0.86 |
| **All AFRICA** | **7.8** | **554** | 70.8 | 87.9 | 0.77 | 0.71 | 0.84 | **25** | 3.2 | 3.4 | 0.91 | 0.61 | 1.34 | **76** | | 9.7 | 10.3 | 2.44 | 1.93 | 3.07 | **122** | 15.6 | 18.7 | 0.95 | 0.79 | 1.14 |
| Iraq | **7.3** | **518** | 71.3 | 85.7 | 0.65 | 0.60 | 0.71 | **30** | 4.1 | 4.8 | 0.94 | 0.66 | 1.36 | **14** | | 1.9 | 2.5 | 0.39 | 0.23 | 0.66 | **147** | 20.2 | 25.2 | 1.07 | 0.91 | 1.27 |
| Syria | **2.0** | **201** | 100.8 | 94.1 | 0.69 | 0.60 | 0.80 | **14** | 7.0 | 7.0 | 1.07 | 0.62 | 1.85 | **8** | | 4.0 | 3.9 | 0.65 | 0.33 | 1.31 | **76** | 38.1 | 35.0 | 1.46 | 1.17 | 1.84 |
| Lebanon | **2.4** | **174** | 72.2 | 99.4 | 0.73 | 0.63 | 0.85 | **9** | 3.7 | 4.3 | 0.81 | 0.40 | 1.62 | **7** | | 2.9 | 4.3 | 0.69 | 0.33 | 1.46 | **60** | 24.9 | 38.8 | 1.44 | 1.12 | 1.86 |
| Iran | **6.1** | **434** | 71.2 | 63.4 | 0.48 | 0.44 | 0.53 | **32** | 5.2 | 4.6 | 0.91 | 0.64 | 1.29 | **22** | | 3.6 | 3.1 | 0.57 | 0.37 | 0.87 | **79** | 13.0 | 11.9 | 0.50 | 0.40 | 0.63 |
| **All Middle EAST** | **17.8** | **1,327** | 74.7 | 79.1 | 0.60 | 0.57 | 0.63 | **85** | 4.8 | 5.1 | 0.93 | 0.75 | 1.16 | **51** | | 2.9 | 3.1 | 0.52 | 0.39 | 0.69 | **362** | 20.4 | 22.4 | 0.92 | 0.83 | 1.02 |
| India | **1.1** | **71** | 67.1 | 67.7 | 0.56 | 0.45 | 0.71 | **2** | 1.9 | 2.2 |  |  |  | **3** | | 2.8 | 3.0 |  |  |  | **10** | 9.4 | 10.2 | 0.51 | 0.28 | 0.95 |
| Vietnam | **1.1** | **104** | 96.3 | 101.4 | 0.75 | 0.62 | 0.92 | **4** | 3.7 | 4.0 |  |  |  | **30** | | 27.8 | 25.8 | 5.20 | 3.62 | 7.46 | **25** | 23.1 | 27.2 | 0.98 | 0.66 | 1.46 |
| Thailand | **0.5** | **23** | 43.7 | 131.0 | 1.18 | 0.77 | 1.79 | **1** | 1.9 | 1.4 |  |  |  | **6** | | 11.4 | 18.3 | 7.04 | 3.15 | 15.71 | **6** | 11.4 | 51.0 | 2.11 | 0.95 | 4.70 |
| China | **0.9** | **123** | 130.5 | 87.7 | 0.58 | 0.49 | 0.70 | **8** | 8.5 | 6.0 | 0.93 | 0.46 | 1.86 | **20** | | 21.2 | 16.0 | 2.63 | 1.69 | 4.08 | **24** | 25.5 | 18.8 | 0.71 | 0.47 | 1.07 |
| **All ASIA** | **8.7** | **650** | 74.4 | 86.3 | 0.66 | 0.61 | 0.71 | **35** | 4.0 | 4.4 | 0.88 | 0.63 | 1.24 | **88** | | 10.1 | 11.0 | 2.25 | 1.82 | 2.79 | **130** | 14.9 | 18.4 | 0.78 | 0.66 | 0.93 |

…continued (sTable 1)

| Countries/region | **Esophageal** | | | | | | | **Laryngeal** | | | | | | **Kidney** | | | | | | **Bladder** | | | | | | **Colorectal** | | | | | |
| --- | --- | --- | --- | --- | --- | --- | --- | --- | --- | --- | --- | --- | --- | --- | --- | --- | --- | --- | --- | --- | --- | --- | --- | --- | --- | --- | --- | --- | --- | --- | --- |
|  | Person years in 100,000 | Total deaths | CMR per 100,000 | ASMR per 100,000 | Hazard ratio, 95% CI | | | Total deaths | CMR per 100,000 | ASMR per 100,000 | Hazard ratio, 95% CI | | | Total deaths | CMR per 100,000 | ASMR per 100,000 | Hazard ratio, 95% CI | | | Total deaths | CMR per 100,000 | ASMR per 100,000 | Hazard ratio, 95% CI | | | Total deaths | CMR per 100,000 | ASMR per 100,000 | Hazard ratio, 95% CI | | |
| **SWEDEN (ref)** | **717.6** | **6,212** | 8.7 | 3.0 | 1.00 |  |  | **960** | 1.3 | 0.4 | 1.00 |  |  | **8,369** | 11.7 | 3.8 | 1.00 |  |  | **9,998** | 13.9 | 3.7 | 1.00 |  |  | **29,426** | 41.0 | 12.5 | 1.00 |  |  |
| **IMMIGRANTS** | **106.4** | **588** | 5.5 | 2.6 | 0.76 | 0.69 | 0.83 | **180** | 1.7 | 0.8 | 1.47 | 1.24 | 1.75 | **830** | 7.8 | 3.6 | 0.92 | 0.85 | 0.99 | **905** | 8.5 | 3.8 | 0.94 | 0.87 | 1.01 | **2,739** | 25.7 | 11.9 | 0.87 | 0.83 | 0.90 |
| Finland | **16.3** | **190** | 11.7 | 3.5 | 1.04 | 0.90 | 1.20 | **45** | 2.8 | 0.8 | 1.61 | 1.18 | 2.19 | **253** | 15.5 | 4.7 | 1.18 | 1.04 | 1.34 | **155** | 9.5 | 2.8 | 0.73 | 0.62 | 0.85 | **611** | 37.5 | 11.3 | 0.88 | 0.81 | 0.95 |
| Denmark | **4.2** | **78** | 18.5 | 4.6 | 1.33 | 1.06 | 1.67 | **14** | 3.3 | 0.8 | 1.44 | 0.85 | 2.44 | **61** | 14.5 | 3.2 | 0.83 | 0.64 | 1.07 | **140** | 33.2 | 6.8 | 1.63 | 1.37 | 1.92 | **274** | 64.9 | 13.7 | 1.05 | 0.93 | 1.19 |
| Norway | **3.4** | **39** | 11.3 | 2.9 | 0.78 | 0.56 | 1.08 | **11** | 3.2 | 0.8 | 1.12 | 0.58 | 2.17 | **69** | 20.0 | 5.2 | 1.16 | 0.91 | 1.48 | **76** | 22.1 | 4.4 | 0.99 | 0.78 | 1.26 | **244** | 70.8 | 15.6 | 1.15 | 1.01 | 1.31 |
| **All NORDIC** | **24.2** | **307** | 12.7 | 3.7 | 1.05 | 0.94 | 1.18 | **70** | 2.9 |  | 1.47 | 1.14 | 1.89 | **386** | 15.9 | 4.4 | 1.10 | 0.99 | 1.23 | **371** | 15.3 | 3.9 | 0.98 | 0.89 | 1.09 | **1,134** | 46.8 | 12.5 | 0.97 | 0.91 | 1.03 |
| Turkey | **4.0** | **4** | 1.0 | 0.8 |  |  |  | **4** | 1.0 | 0.7 |  |  |  | **11** | 2.7 | 2.2 | 0.46 | 0.26 | 0.83 | **13** | 3.2 | 2.5 | 0.50 | 0.28 | 0.88 | **41** | 10.2 | 7.5 | 0.48 | 0.35 | 0.65 |
| Germany | **3.8** | **48** | 12.6 | 2.9 | 1.02 | 0.76 | 1.35 | **8** | 2.1 | 0.8 | 1.18 | 0.58 | 2.36 | **66** | 17.3 | 3.8 | 1.12 | 0.88 | 1.43 | **76** | 20.0 | 4.2 | 1.06 | 0.84 | 1.34 | **232** | 60.9 | 13.9 | 1.06 | 0.93 | 1.21 |
| Austria | **0.7** | **7** | 9.5 | 1.9 | 0.74 | 0.35 | 1.55 | **1** | 1.4 | 0.3 |  |  |  | **5** | 6.8 | 1.1 | 0.42 | 0.18 | 1.01 | **7** | 9.5 | 1.8 | 0.52 | 0.25 | 1.08 | **48** | 64.9 | 14.0 | 1.13 | 0.85 | 1.50 |
| Netherlands | **0.6** | **5** | 8.1 | 3.3 | 0.87 | 0.36 | 2.09 | **1** | 1.6 | 0.3 |  |  |  | **6** | 9.7 | 3.9 | 0.87 | 0.39 | 1.94 | **10** | 16.1 | 5.2 | 1.31 | 0.70 | 2.43 | **18** | 29.1 | 10.1 | 0.73 | 0.46 | 1.16 |
| France | **0.6** | **3** | 4.8 | 2.9 |  |  |  | **0** |  |  |  |  |  | **5** | 7.9 | 4.4 | 0.97 | 0.37 | 2.59 | **4** | 6.3 | 3.4 |  |  |  | **10** | 15.9 | 6.9 | 0.67 | 0.36 | 1.25 |
| For. Yugoslavia | **7.1** | **24** | 3.4 | 1.6 | 0.43 | 0.28 | 0.65 | **24** | 3.4 | 1.6 | 2.99 | 1.98 | 4.52 | **56** | 7.9 | 3.9 | 0.97 | 0.74 | 1.26 | **58** | 8.2 | 4.2 | 1.05 | 0.81 | 1.36 | **176** | 24.9 | 12.9 | 0.90 | 0.78 | 1.04 |
| Bosnian-Hercegovina | **4.8** | **10** | 2.1 | 1.4 | 0.35 | 0.19 | 0.66 | **12** | 2.5 | 1.7 | 2.61 | 1.44 | 4.75 | **19** | 4.0 | 2.7 | 0.67 | 0.42 | 1.05 | **32** | 6.7 | 4.6 | 1.14 | 0.80 | 1.62 | **85** | 17.7 | 11.9 | 0.80 | 0.65 | 0.99 |
| Italy | **0.9** | **7** | 7.5 | 2.2 | 0.66 | 0.31 | 1.38 | **4** | 4.3 | 1.0 |  |  |  | **11** | 11.8 | 3.1 | 0.86 | 0.47 | 1.54 | **21** | 22.5 | 5.8 | 1.40 | 0.91 | 2.18 | **39** | 41.7 | 11.6 | 0.85 | 0.62 | 1.17 |
| Spain | **0.6** | **4** | 6.2 | 2.6 |  |  |  | **2** | 3.1 | 1.2 |  |  |  | **5** | 7.7 | 3.2 | 0.87 | 0.36 | 2.09 | **10** | 15.5 | 6.0 | 1.79 | 0.96 | 3.32 | **17** | 26.3 | 12.0 | 0.87 | 0.54 | 1.40 |
| Greece | **1.4** | **1** | 0.7 | 0.3 |  |  |  | **1** | 0.7 | 0.3 |  |  |  | **7** | 5.1 | 2.0 | 0.48 | 0.23 | 1.01 | **13** | 9.5 | 4.0 | 0.97 | 0.57 | 1.68 | **35** | 25.6 | 10.3 | 0.71 | 0.51 | 0.99 |
| Croatia | **0.6** | **0** | 0.0 |  |  |  |  | **2** | 3.4 | 1.4 |  |  |  | **4** | 6.8 | 2.7 |  |  |  | **5** | 8.5 | 6.9 | 1.13 | 0.47 | 2.71 | **12** | 20.4 | 12.4 | 0.74 | 0.42 | 1.30 |
| Poland | **3.9** | **16** | 4.1 | 1.9 | 0.60 | 0.37 | 0.98 | **11** | 2.8 | 1.5 | 2.59 | 1.42 | 4.72 | **35** | 9.1 | 4.6 | 1.12 | 0.80 | 1.56 | **38** | 9.8 | 4.2 | 0.98 | 0.70 | 1.36 | **99** | 25.6 | 11.8 | 0.86 | 0.71 | 1.05 |
| Hungary | **1.6** | **11** | 6.7 | 1.4 | 0.53 | 0.29 | 0.96 | **8** | 4.9 | 1.2 | 2.62 | 1.31 | 5.27 | **30** | 18.3 | 4.8 | 1.11 | 0.76 | 1.60 | **33** | 20.1 | 4.3 | 1.18 | 0.84 | 1.67 | **85** | 51.8 | 12.8 | 0.96 | 0.78 | 1.19 |
| Fo. Czechoslovakia | **0.7** | **4** | 5.6 | 1.4 |  |  |  | **5** | 7.0 | 1.7 | 3.05 | 1.14 | 8.15 | **14** | 19.7 | 4.0 | 1.06 | 0.61 | 1.82 | **14** | 19.7 | 3.8 | 0.96 | 0.57 | 1.63 | **57** | 80.4 | 18.4 | 1.29 | 0.99 | 1.68 |
| Romania | **1.4** | **3** | 2.2 | 1.4 |  |  |  | **3** | 2.2 | 1.0 |  |  |  | **5** | 3.7 | 2.0 | 0.45 | 0.19 | 1.08 | **7** | 5.2 | 2.3 | 0.57 | 0.27 | 1.21 | **24** | 17.7 | 8.9 | 0.59 | 0.40 | 0.88 |
| For. Sovjetunionen | **0.6** | **9** | 16.2 | 2.7 | 1.15 | 0.60 | 2.21 | **1** | 1.8 | 0.6 |  |  |  | **14** | 25.2 | 5.6 | 1.29 | 0.76 | 2.18 | **13** | 23.4 | 3.8 | 0.90 | 0.52 | 1.54 | **41** | 73.7 | 15.2 | 0.93 | 0.67 | 1.29 |
| Russia | **0.4** | **2** | 4.5 | 5.9 |  |  |  | **0** |  |  |  |  |  | **0** |  |  |  |  |  | **1** | 2.2 | 1.5 |  |  |  | **7** | 15.6 | 20.0 | 1.14 | 0.54 | 2.39 |
| Estonia | **1.0** | **20** | 20.5 | 5.3 | 1.14 | 0.73 | 1.77 | **3** | 3.1 | 0.3 |  |  |  | **35** | 35.9 | 3.8 | 1.22 | 0.86 | 1.73 | **41** | 42.0 | 4.0 | 1.14 | 0.84 | 1.55 | **120** | 123.1 | 15.8 | 1.18 | 0.99 | 1.42 |
| UK & North Ireland | **2.0** | **19** | 9.5 | 5.7 | 1.62 | 1.03 | 2.54 | **1** | 0.5 | 0.3 |  |  |  | **7** | 3.5 | 2.0 | 0.56 | 0.27 | 1.17 | **12** | 6.0 | 3.8 | 0.97 | 0.55 | 1.71 | **48** | 24.0 | 14.8 | 1.04 | 0.78 | 1.38 |
| **All Non-NORDIC EUROPÉ** | **40.0** | **206** | 5.1 | 2.1 | 0.63 | 0.54 | 0.72 | **95** | 2.4 |  | 1.89 | 1.51 | 2.36 | **348** | 8.7 | 3.5 | 0.89 | 0.80 | 1.00 | **429** | 10.7 | 4.0 | 1.00 | 0.91 | 1.11 | **1,259** | 31.4 | 12.6 | 0.91 | 0.86 | 0.97 |
| USA | **1.4** | **11** | 8.0 | 2.5 | 0.91 | 0.51 | 1.65 | **1** | 0.7 | 0.2 |  |  |  | **11** | 8.0 | 2.7 | 0.69 | 0.38 | 1.24 | **25** | 18.2 | 4.3 | 1.24 | 0.83 | 1.83 | **46** | 33.5 | 8.9 | 0.78 | 0.58 | 1.04 |
| **All North AMERICA** | **2.4** | **12** | 5.0 | 2.1 | 0.76 | 0.43 | 1.33 | **1** | 0.4 |  |  |  |  | **13** | 5.4 | 2.5 | 0.60 | 0.34 | 1.05 | **29** | 12.0 | 3.8 | 1.19 | 0.82 | 1.71 | **51** | 21.1 | 8.3 | 0.68 | 0.52 | 0.90 |
| Chile | **2.8** | **6** | 2.2 | 1.7 | 0.41 | 0.18 | 0.91 | **2** | 0.7 | 0.6 |  |  |  | **11** | 4.0 | 3.3 | 0.75 | 0.42 | 1.36 | **5** | 1.8 | 1.9 | 0.36 | 0.15 | 0.87 | **27** | 9.7 | 7.9 | 0.53 | 0.37 | 0.78 |
| **All South AMERICA** | **5.0** | **11** | 2.2 | 2.0 | 0.48 | 0.26 | 0.86 | **2** | 0.4 |  |  |  |  | **19** | 3.8 | 3.3 | 0.82 | 0.52 | 1.29 | **7** | 1.4 | 1.5 | 0.32 | 0.15 | 0.67 | **52** | 10.4 | 9.1 | 0.63 | 0.48 | 0.82 |
| Ethiopia | **1.2** | **2** | 1.6 | 2.0 |  |  |  | **0** |  |  |  |  |  | **4** | 3.3 | 7.2 |  |  |  | **1** | 0.8 | 1.8 |  |  |  | **5** | 4.1 | 6.1 | 0.47 | 0.20 | 1.14 |
| Somalia | **1.8** | **2** | 1.1 | 2.4 |  |  |  | **1** | 0.6 | 0.6 |  |  |  | **4** | 2.2 | 3.2 |  |  |  | **1** | 0.6 | 2.2 |  |  |  | **2** | 1.1 | 1.8 |  |  |  |
| **All AFRICA** | **7.8** | **11** | 1.4 | 1.5 | 0.45 | 0.25 | 0.82 | **5** | 0.6 |  | 1.36 | 0.56 | 3.30 | **14** | 1.8 | 2.1 | 0.60 | 0.35 | 1.01 | **9** | 1.1 | 1.3 | 0.46 | 0.24 | 0.89 | **40** | 5.1 | 6.9 | 0.47 | 0.34 | 0.64 |
| Iraq | **7.3** | **6** | 0.8 | 1.0 | 0.22 | 0.10 | 0.50 | **2** | 0.3 | 0.2 |  |  |  | **24** | 3.3 | 4.0 | 0.95 | 0.63 | 1.43 | **15** | 2.1 | 2.5 | 0.62 | 0.36 | 1.04 | **48** | 6.6 | 7.2 | 0.50 | 0.38 | 0.67 |
| Syria | **2.0** | **4** | 2.0 | 1.9 |  |  |  | **0** |  |  |  |  |  | **5** | 2.5 | 2.4 | 0.52 | 0.22 | 1.25 | **9** | 4.5 | 4.1 | 1.01 | 0.53 | 1.95 | **11** | 5.5 | 4.9 | 0.32 | 0.18 | 0.58 |
| Lebanon | **2.4** | **1** | 0.4 | 0.2 |  |  |  | **0** |  |  |  |  |  | **4** | 1.7 | 2.4 |  |  |  | **8** | 3.3 | 6.5 | 1.22 | 0.61 | 2.44 | **12** | 5.0 | 5.7 | 0.44 | 0.25 | 0.77 |
| Iran | **6.1** | **10** | 1.6 | 1.2 | 0.34 | 0.18 | 0.64 | **2** | 0.3 | 0.3 |  |  |  | **3** | 0.5 | 0.5 |  |  |  | **10** | 1.6 | 1.9 | 0.32 | 0.17 | 0.62 | **59** | 9.7 | 8.2 | 0.56 | 0.44 | 0.73 |
| **All Middle EAST** | **17.8** | **21** | 1.2 | 1.1 | 0.29 | 0.19 | 0.45 | **4** | 0.2 |  |  |  |  | **36** | 2.0 | 2.1 | 0.50 | 0.36 | 0.70 | **42** | 2.4 | 2.8 | 0.61 | 0.44 | 0.83 | **130** | 7.3 | 7.1 | 0.50 | 0.42 | 0.60 |
| India | **1.1** | **3** | 2.8 | 2.7 |  |  |  | **0** |  |  |  |  |  | **1** | 0.9 | 0.8 |  |  |  | **1** | 0.9 | 1.1 |  |  |  | **9** | 8.5 | 9.1 | 0.61 | 0.32 | 1.17 |
| Vietnam | **1.1** | **3** | 2.8 | 4.4 |  |  |  | **0** |  |  |  |  |  | **1** | 0.9 | 1.2 |  |  |  | **1** | 0.9 | 1.4 |  |  |  | **8** | 7.4 | 8.4 | 0.51 | 0.26 | 1.02 |
| Thailand | **0.5** | **0** | 0.0 |  |  |  |  | **0** |  |  |  |  |  | **1** | 1.9 | 1.4 |  |  |  | **0** | 0.0 |  |  |  |  | **3** | 5.7 | 30.6 |  |  |  |
| China | **0.9** | **1** | 1.1 | 1.1 |  |  |  | **2** | 2.1 | 0.9 |  |  |  | **3** | 3.2 | 2.1 |  |  |  | **2** | 2.1 | 0.8 |  |  |  | **12** | 12.7 | 8.3 | 0.50 | 0.28 | 0.87 |
| **All ASIA** | **8.7** | **18** | 2.1 | 2.5 | 0.60 | 0.38 | 0.96 | **3** | 0.3 |  |  |  |  | **13** | 1.5 | 1.8 | 0.38 | 0.22 | 0.68 | **18** | 2.1 | 2.7 | 0.60 | 0.38 | 0.96 | **71** | 8.1 | 9.1 | 0.62 | 0.49 | 0.78 |

...continued (sTable 1)

| Countries/region | **Pancreas** | | | | | | | **Prostate** | | | | | | **Brain and CNS** | | | | | | **Malignant Melanoma** | | | | | |
| --- | --- | --- | --- | --- | --- | --- | --- | --- | --- | --- | --- | --- | --- | --- | --- | --- | --- | --- | --- | --- | --- | --- | --- | --- | --- |
|  | Person years in 100,000 | Total deaths | CMR per 100,000 | ASMR per 100,000 | Hazard ratio, 95% CI | | | Total deaths | CMR per 100,000 | ASMR per 100,000 | Hazard ratio, 95% CI | | | Total deaths | CMR per 100,000 | ASMR per 100,000 | Hazard ratio, 95% CI | | | Total deaths | CMR per 100,000 | ASMR per 100,000 | Hazard ratio, 95% CI | | |
| **SWEDEN (ref)** | **717.6** | **15,682** | 21.9 | 7.2 | 1.00 |  |  | **55,795** | 77.7 | 18.8 | 1.00 |  |  | **8,262** | 11.5 | 4.9 | 1.00 |  |  | **5,608** | 7.8 | 2.9 | 1.00 |  |  |
| **IMMIGRANTS** | **106.4** | **1,682** | 15.8 | 7.4 | 0.93 | 0.88 | 0.98 | **3,371** | 31.7 | 13.6 | 0.70 | 0.68 | 0.73 | **841** | 7.9 | 4.0 | 0.74 | 0.68 | 0.79 | **392** | 3.7 | 1.8 | 0.57 | 0.51 | 0.63 |
| Finland | **16.3** | **460** | 28.3 | 8.3 | 1.11 | 1.01 | 1.22 | **920** | 56.5 | 16.2 | 0.90 | 0.84 | 0.96 | **191** | 11.7 | 4.4 | 0.82 | 0.71 | 0.95 | **103** | 6.3 | 2.2 | 0.65 | 0.53 | 0.80 |
| Denmark | **4.2** | **151** | 35.8 | 8.7 | 1.03 | 0.87 | 1.22 | **392** | 92.9 | 16.9 | 0.84 | 0.76 | 0.93 | **62** | 14.7 | 4.6 | 0.90 | 0.69 | 1.16 | **43** | 10.2 | 2.8 | 0.93 | 0.69 | 1.27 |
| Norway | **3.4** | **108** | 31.3 | 7.2 | 0.91 | 0.75 | 1.11 | **354** | 102.7 | 17.2 | 0.89 | 0.80 | 0.99 | **37** | 10.7 | 3.3 | 0.64 | 0.45 | 0.90 | **35** | 10.2 | 2.9 | 0.94 | 0.67 | 1.32 |
| **All NORDIC** | **24.2** | **7,221** | 298.1 | 8.2 | 1.06 | 0.98 | 1.14 | **1,672** | 69.0 | 16.6 | 0.88 | 0.84 | 0.93 | **291** | 12.0 | 4.3 | 0.81 | 0.71 | 0.91 | **182** | 7.5 | 2.4 | 0.76 | 0.65 | 0.88 |
| Turkey | **4.0** | **37** | 9.2 | 6.9 | 0.76 | 0.55 | 1.06 | **25** | 6.2 | 4.8 | 0.22 | 0.15 | 0.33 | **24** | 6.0 | 4.1 | 0.65 | 0.43 | 0.98 | **2** | 0.5 | 0.3 |  |  |  |
| Germany | **3.8** | **116** | 30.5 | 6.4 | 0.96 | 0.80 | 1.15 | **289** | 75.9 | 14.7 | 0.76 | 0.68 | 0.86 | **58** | 15.2 | 4.5 | 0.95 | 0.73 | 1.22 | **25** | 6.6 | 1.6 | 0.59 | 0.40 | 0.88 |
| Austria | **0.7** | **29** | 39.2 | 8.6 | 1.21 | 0.84 | 1.74 | **52** | 70.3 | 12.7 | 0.70 | 0.53 | 0.92 | **15** | 20.3 | 4.9 | 1.21 | 0.73 | 2.00 | **11** | 14.9 | 3.3 | 1.27 | 0.70 | 2.30 |
| Netherlands | **0.6** | **21** | 33.9 | 12.0 | 1.49 | 0.97 | 2.29 | **33** | 53.3 | 14.3 | 0.80 | 0.56 | 1.13 | **8** | 12.9 | 6.1 | 0.98 | 0.49 | 1.97 | **3** | 4.8 | 1.7 |  |  |  |
| France | **0.6** | **6** | 9.5 | 6.0 | 0.60 | 0.25 | 1.43 | **25** | 39.6 | 20.4 | 1.01 | 0.68 | 1.51 | **14** | 22.2 | 12.8 | 2.43 | 1.44 | 4.10 | **2** | 3.2 | 1.8 |  |  |  |
| For. Yugoslavia | **7.1** | **97** | 13.7 | 6.6 | 0.82 | 0.67 | 1.00 | **105** | 14.9 | 8.8 | 0.42 | 0.35 | 0.51 | **77** | 10.9 | 5.9 | 1.00 | 0.80 | 1.26 | **21** | 3.0 | 1.5 | 0.48 | 0.31 | 0.73 |
| Bosnian-Hercegovina | **4.8** | **44** | 9.2 | 6.6 | 0.71 | 0.53 | 0.96 | **44** | 9.2 | 7.1 | 0.36 | 0.26 | 0.48 | **39** | 8.1 | 4.9 | 0.93 | 0.67 | 1.28 | **17** | 3.5 | 2.6 | 0.73 | 0.45 | 1.18 |
| Italy | **0.9** | **24** | 25.7 | 7.3 | 0.91 | 0.60 | 1.36 | **38** | 40.6 | 9.8 | 0.52 | 0.38 | 0.72 | **9** | 9.6 | 2.8 | 0.66 | 0.34 | 1.27 | **3** | 3.2 | 0.8 |  |  |  |
| Spain | **0.6** | **12** | 18.6 | 7.7 | 1.04 | 0.59 | 1.83 | **13** | 20.1 | 8.7 | 0.49 | 0.29 | 0.85 | **3** | 4.6 | 5.9 |  |  |  | **1** | 1.5 | 0.5 |  |  |  |
| Greece | **1.4** | **21** | 15.4 | 5.6 | 0.73 | 0.48 | 1.12 | **16** | 11.7 | 5.0 | 0.26 | 0.16 | 0.43 | **5** | 3.7 | 1.4 | 0.27 | 0.11 | 0.65 | **1** | 0.7 | 0.3 |  |  |  |
| Croatia | **0.6** | **9** | 15.3 | 9.7 | 0.90 | 0.47 | 1.73 | **8** | 13.6 | 5.6 | 0.42 | 0.21 | 0.84 | **2** | 3.4 | 1.6 |  |  |  | **3** | 5.1 | 5.4 |  |  |  |
| Poland | **3.9** | **61** | 15.8 | 7.1 | 0.94 | 0.73 | 1.21 | **101** | 26.1 | 11.0 | 0.53 | 0.43 | 0.64 | **26** | 6.7 | 4.0 | 0.62 | 0.42 | 0.91 | **12** | 3.1 | 1.7 | 0.49 | 0.28 | 0.86 |
| Hungary | **1.6** | **52** | 31.7 | 8.7 | 1.03 | 0.78 | 1.35 | **103** | 62.8 | 13.8 | 0.70 | 0.57 | 0.85 | **15** | 9.1 | 2.5 | 0.51 | 0.30 | 0.86 | **16** | 9.7 | 3.0 | 0.89 | 0.55 | 1.46 |
| For. Czechoslovakia | **0.7** | **24** | 33.8 | 7.0 | 0.99 | 0.67 | 1.48 | **55** | 77.5 | 13.7 | 0.66 | 0.50 | 0.86 | **7** | 9.9 | 4.3 | 0.56 | 0.27 | 1.18 | **4** | 5.6 | 1.6 |  |  |  |
| Romania | **1.4** | **21** | 15.5 | 8.3 | 0.92 | 0.60 | 1.41 | **26** | 19.2 | 8.9 | 0.43 | 0.29 | 0.63 | **8** | 5.9 | 3.2 | 0.54 | 0.27 | 1.08 | **6** | 4.4 | 2.4 | 0.58 | 0.24 | 1.40 |
| For. Sovjetunionen | **0.6** | **16** | 28.8 | 6.8 | 0.69 | 0.41 | 1.17 | **54** | 97.1 | 12.6 | 0.62 | 0.47 | 0.81 | **4** | 7.2 | 2.9 |  |  |  | **4** | 7.2 | 1.6 |  |  |  |
| Russia | **0.4** | **5** | 11.1 | 11.5 | 1.50 | 0.62 | 3.60 | **2** | 4.5 | 5.0 |  |  |  | **2** | 4.5 | 2.2 |  |  |  | **2** | 4.5 | 2.5 |  |  |  |
| Estonia | **1.0** | **54** | 55.4 | 8.6 | 1.07 | 0.82 | 1.41 | **216** | 221.5 | 19.9 | 0.96 | 0.84 | 1.10 | **25** | 25.6 | 2.9 | 1.15 | 0.77 | 1.74 | **20** | 20.5 | 3.2 | 1.26 | 0.81 | 1.96 |
| UK & North Ireland | **2.0** | **16** | 8.0 | 5.0 | 0.60 | 0.36 | 0.97 | **36** | 18.0 | 12.1 | 0.57 | 0.40 | 0.79 | **18** | 9.0 | 5.4 | 0.94 | 0.59 | 1.50 | **5** | 2.5 | 1.2 | 0.36 | 0.13 | 0.96 |
| **All Non-NORDIC EUROPÉ** | **40.0** | **698** | 17.4 | 7.0 | 0.90 | 0.83 | 0.97 | **1,343** | 33.5 | 12.1 | 0.61 | 0.58 | 0.65 | **377** | 9.4 | 4.3 | 0.80 | 0.72 | 0.89 | **170** | 4.2 | 1.8 | 0.59 | 0.50 | 0.69 |
| USA | **1.4** | **35** | 25.5 | 8.5 | 1.10 | 0.79 | 1.54 | **107** | 77.9 | 17.2 | 0.87 | 0.72 | 1.06 | **17** | 12.4 | 4.8 | 0.99 | 0.62 | 1.60 | **11** | 8.0 | 4.0 | 0.97 | 0.54 | 1.75 |
| **All North AMERICA** | **2.4** | **43** | 17.8 | 8.4 | 1.04 | 0.77 | 1.41 | **126** | 52.2 | 17.5 | 0.88 | 0.74 | 1.06 | **25** | 10.4 | 3.8 | 0.82 | 0.53 | 1.27 | **11** | 4.6 | 2.6 | 0.72 | 0.40 | 1.30 |
| Chile | **2.8** | **30** | 10.8 | 8.8 | 0.97 | 0.67 | 1.39 | **32** | 11.5 | 11.9 | 0.51 | 0.36 | 0.73 | **7** | 2.5 | 2.1 | 0.26 | 0.12 | 0.58 | **4** | 1.4 | 1.2 |  |  |  |
| **All South AMERICA** | **5.0** | **47** | 9.4 | 8.3 | 0.96 | 0.72 | 1.28 | **54** | 10.8 | 12.1 | 0.53 | 0.41 | 0.70 | **20** | 4.0 | 2.1 | 0.31 | 0.18 | 0.55 | **8** | 1.6 | 1.3 | 0.34 | 0.16 | 0.72 |
| Ethiopia | **1.2** | **6** | 4.9 | 10.4 |  |  |  | **1** | 0.8 | 4.9 |  |  |  | **4** | 3.3 | 2.8 |  |  |  | **0** |  |  |  |  |  |
| Somalia | **1.8** | **4** | 2.2 | 3.9 | 0.54 | 0.20 | 1.45 | **2** | 1.1 | 2.2 |  |  |  | **1** | 0.6 | 1.4 |  |  |  | **0** |  |  |  |  |  |
| **All AFRICA** | **7.8** | **52** | 6.6 | 7.9 | 1.02 | 0.77 | 1.34 | **42** | 5.4 | 12.1 | 0.55 | 0.41 | 0.75 | **24** | 3.1 | 2.8 | 0.44 | 0.29 | 0.67 | **4** | 0.5 | 0.6 |  |  |  |
| Iraq | **7.3** | **36** | 5.0 | 6.7 | 0.64 | 0.46 | 0.90 | **34** | 4.7 | 8.1 | 0.38 | 0.27 | 0.53 | **34** | 4.7 | 4.5 | 0.63 | 0.44 | 0.88 | **1** | 0.1 | 0.4 |  |  |  |
| Syria | **2.0** | **9** | 4.5 | 4.5 | 0.45 | 0.24 | 0.87 | **11** | 5.5 | 6.5 | 0.29 | 0.16 | 0.52 | **9** | 4.5 | 4.3 | 0.55 | 0.28 | 1.05 | **3** | 1.5 | 1.1 |  |  |  |
| Lebanon | **2.4** | **15** | 6.2 | 7.9 | 0.88 | 0.52 | 1.49 | **7** | 2.9 | 6.4 | 0.27 | 0.13 | 0.56 | **10** | 4.2 | 3.6 | 0.63 | 0.34 | 1.17 | **5** | 2.1 | 1.1 | 0.65 | 0.27 | 1.56 |
| Iran | **6.1** | **24** | 3.9 | 3.5 | 0.37 | 0.25 | 0.56 | **35** | 5.7 | 6.5 | 0.29 | 0.21 | 0.41 | **35** | 5.7 | 4.3 | 0.69 | 0.49 | 0.96 | **2** | 0.3 | 0.2 |  |  |  |
| **All Middle EAST** | **17.8** | **84** | 4.7 | 5.2 | 0.54 | 0.43 | 0.67 | **87** | 4.9 | 7.0 | 0.32 | 0.26 | 0.39 | **88** | 5.0 | 4.3 | 0.64 | 0.52 | 0.80 | **11** | 0.6 | 0.5 | 0.16 | 0.09 | 0.29 |
| India | **1.1** | **5** | 4.7 | 4.8 | 0.58 | 0.24 | 1.39 | **3** | 2.8 | 3.0 |  |  |  | **5** | 4.7 | 3.4 | 0.68 | 0.28 | 1.62 | **0** |  |  |  |  |  |
| Vietnam | **1.1** | **3** | 2.8 | 2.2 |  |  |  | **5** | 4.6 | 6.0 | 0.27 | 0.11 | 0.65 | **2** | 1.9 | 1.7 |  |  |  | **0** |  |  |  |  |  |
| Thailand | **0.5** | **0** | 0.0 |  |  |  |  | **1** | 1.9 | 22.2 |  |  |  | **1** | 1.9 | 0.7 |  |  |  | **0** |  |  |  |  |  |
| China | **0.9** | **7** | 7.4 | 5.9 | 0.55 | 0.26 | 1.14 | **13** | 13.8 | 6.3 | 0.30 | 0.17 | 0.53 | **5** | 5.3 | 4.0 | 0.55 | 0.23 | 1.32 | **2** | 2.1 | 1.0 |  |  |  |
| **All ASIA** | **8.7** | **36** | 4.1 | 4.8 | 0.54 | 0.39 | 0.75 | **44** | 5.0 | 6.8 | 0.31 | 0.23 | 0.41 | **25** | 2.9 | 2.7 | 0.40 | 0.27 | 0.60 | **2** | 0.2 | 0.4 |  |  |  |

…continued (sTable 1)

| Countries/region | **Mesothelioma** | | | | | | | **Hematological** | | | | | | **Gall-bladder** | | | | | | **Head and neck cancer** | | | | | |
| --- | --- | --- | --- | --- | --- | --- | --- | --- | --- | --- | --- | --- | --- | --- | --- | --- | --- | --- | --- | --- | --- | --- | --- | --- | --- |
|  | Person years in 100,000 | Total deaths | CMR per 100,000 | ASMR per 100,000 | Hazard ratio, 95% CI | | | Total deaths | CMR per 100,000 | ASMR per 100,000 | Hazard ratio, 95% CI | | | Total deaths | CMR per 100,000 | ASMR per 100,000 | Hazard ratio, 95% CI | | | Total deaths | CMR per 100,000 | ASMR per 100,000 | Hazard ratio, 95% CI | | |
| **SWEDEN (ref)** | **717.6** | **1,851** | 2.6 | 0.9 | 1.00 |  |  | **19,831** | 27.6 | 8.6 | 1.00 |  |  | **1,868** | 2.6 | 0.8 | 1.00 |  |  | **3,656** | 5.1 | 1.9 | 1.00 |  |  |
| **IMMIGRANTS** | **106.4** | **228** | 2.1 | 1.0 | 1.06 | 0.92 | 1.22 | **1,956** | 18.4 | 8.5 | 0.95 | 0.90 | 0.99 | **150** | 1.4 | 0.6 | 0.85 | 0.71 | 1.01 | **462** | 4.3 | 2.1 | 0.84 | 0.76 | 0.94 |
| Finland | **16.3** | **65** | 4.0 | 1.1 | 1.19 | 0.93 | 1.53 | **442** | 27.2 | 8.7 | 0.98 | 0.89 | 1.08 | **29** | 1.8 | 0.5 | 0.75 | 0.52 | 1.09 | **144** | 8.8 | 2.9 | 1.21 | 1.02 | 1.43 |
| Denmark | **4.2** | **23** | 5.4 | 1.2 | 1.38 | 0.92 | 2.09 | **194** | 46.0 | 10.6 | 1.17 | 1.02 | 1.35 | **15** | 3.6 | 0.9 | 0.89 | 0.52 | 1.54 | **36** | 8.5 | 2.2 | 0.94 | 0.68 | 1.31 |
| Norway | **3.4** | **17** | 4.9 | 1.1 | 1.32 | 0.82 | 2.13 | **132** | 38.3 | 9.1 | 0.96 | 0.80 | 1.14 | **14** | 4.1 | 1.6 | 1.01 | 0.57 | 1.78 | **26** | 7.5 | 2.0 | 0.80 | 0.54 | 1.19 |
| **All NORDIC** | **24.2** | **106** | 4.4 | 1.2 | 1.26 | 1.03 | 1.53 | **769** | 31.8 | 8.9 | 1.01 | 0.94 | 1.09 | **58** | 2.4 | 0.7 | 0.83 | 0.63 | 1.09 | **207** | 8.5 | 2.6 | 1.09 | 0.94 | 1.25 |
| Turkey | **4.0** | **13** | 3.2 | 2.2 | 2.34 | 1.35 | 4.06 | **51** | 12.7 | 9.6 | 0.93 | 0.70 | 1.23 | **4** | 1.0 | 0.7 | 0.88 | 0.33 | 2.37 | **4** | 1.0 | 0.8 |  |  |  |
| Germany | **3.8** | **19** | 5.0 | 1.2 | 1.22 | 0.78 | 1.92 | **133** | 34.9 | 9.1 | 0.92 | 0.78 | 1.10 | **7** | 1.8 | 0.5 | 0.45 | 0.19 | 1.09 | **31** | 8.1 | 2.5 | 1.00 | 0.70 | 1.43 |
| Austria | **0.7** | **5** | 6.8 | 1.2 | 1.52 | 0.63 | 3.66 | **30** | 40.5 | 8.4 | 1.02 | 0.70 | 1.48 | **0** |  |  |  |  |  | **4** | 5.4 | 1.4 |  |  |  |
| Netherlands | **0.6** | **0** |  |  |  |  |  | **13** | 21.0 | 7.6 | 0.75 | 0.43 | 1.32 | **1** | 1.6 | 0.5 |  |  |  | **1** | 1.6 | 0.6 |  |  |  |
| France | **0.6** | **0** |  |  |  |  |  | **12** | 19.0 | 9.1 | 1.21 | 0.69 | 2.13 | **0** |  |  |  |  |  | **7** | 11.1 | 7.2 | 2.76 | 1.31 | 5.80 |
| For. Yugoslavia | **7.1** | **15** | 2.1 | 1.0 | 0.95 | 0.57 | 1.58 | **117** | 16.6 | 8.3 | 0.93 | 0.78 | 1.12 | **12** | 1.7 | 0.8 | 1.20 | 0.68 | 2.12 | **34** | 4.8 | 2.2 | 0.84 | 0.59 | 1.19 |
| Bosnian-Hercegovina | **4.8** | **4** | 0.8 | 0.7 |  |  |  | **53** | 11.0 | 7.5 | 0.85 | 0.65 | 1.12 | **8** | 1.7 | 1.1 | 1.64 | 0.80 | 3.33 | **15** | 3.1 | 2.0 | 0.68 | 0.41 | 1.14 |
| Italy | **0.9** | **7** | 7.5 | 2.3 | 2.08 | 0.99 | 4.38 | **30** | 32.1 | 7.7 | 1.00 | 0.70 | 1.44 | **2** | 2.1 | 0.5 |  |  |  | **6** | 6.4 | 2.0 | 0.72 | 0.30 | 1.74 |
| Spain | **0.6** | **0** |  |  |  |  |  | **15** | 23.2 | 9.6 | 1.19 | 0.72 | 1.97 | **0** |  |  |  |  |  | **3** | 4.6 | 2.0 |  |  |  |
| Greece | **1.4** | **1** | 0.7 | 0.2 |  |  |  | **34** | 24.9 | 10.6 | 1.04 | 0.74 | 1.48 | **1** | 0.7 | 0.3 |  |  |  | **3** | 2.2 | 0.8 |  |  |  |
| Croatia | **0.6** | **0** |  |  |  |  |  | **10** | 17.0 | 7.3 | 0.98 | 0.53 | 1.82 | **0** |  |  |  |  |  | **1** | 1.7 | 0.9 |  |  |  |
| Poland | **3.9** | **4** | 1.0 | 0.5 |  |  |  | **77** | 19.9 | 9.2 | 1.00 | 0.80 | 1.26 | **4** | 1.0 | 0.6 |  |  |  | **13** | 3.4 | 1.9 | 0.65 | 0.38 | 1.12 |
| Hungary | **1.6** | **7** | 4.3 | 1.2 | 1.06 | 0.50 | 2.22 | **60** | 36.6 | 8.7 | 1.01 | 0.78 | 1.31 | **5** | 3.0 | 0.7 | 1.05 | 0.44 | 2.52 | **10** | 6.1 | 1.6 | 0.72 | 0.39 | 1.34 |
| For. Czechoslovakia | **0.7** | **3** | 4.2 | 1.2 |  |  |  | **38** | 53.6 | 14.6 | 1.20 | 0.86 | 1.67 | **0** |  |  |  |  |  | **11** | 15.5 | 4.4 | 1.90 | 1.05 | 3.44 |
| Romania | **1.4** | **1** | 0.7 | 0.3 |  |  |  | **14** | 10.3 | 5.3 | 0.43 | 0.24 | 0.78 | **3** | 2.2 | 1.3 |  |  |  | **6** | 4.4 | 2.4 | 0.81 | 0.36 | 1.80 |
| For. Sovjetunionen | **0.6** | **2** | 3.6 | 0.6 |  |  |  | **18** | 32.4 | 6.3 | 0.65 | 0.40 | 1.04 | **6** | 10.8 | 1.4 | 1.96 | 0.82 | 4.73 | **3** | 5.4 | 0.6 |  |  |  |
| Russia | **0.4** | **0** |  |  |  |  |  | **2** | 4.5 | 3.1 |  |  |  | **0** |  |  |  |  |  | **0** | 0.0 |  |  |  |  |
| Estonia | **1.0** | **5** | 5.1 | 0.5 | 0.90 | 0.37 | 2.16 | **59** | 60.5 | 10.9 | 0.86 | 0.66 | 1.12 | **11** | 11.3 | 1.4 | 1.63 | 0.88 | 3.04 | **19** | 19.5 | 3.4 | 1.78 | 1.12 | 2.83 |
| UK & North Ireland | **2.0** | **4** | 2.0 | 1.4 |  |  |  | **31** | 15.5 | 9.6 | 1.05 | 0.73 | 1.50 | **0** |  |  |  |  |  | **5** | 2.5 | 1.4 | 0.56 | 0.23 | 1.36 |
| **All Non-NORDIC EUROPÉ** | **40.0** | **99** | 2.5 | 1.1 | 1.04 | 0.85 | 1.29 | **847** | 21.2 | 8.7 | 0.93 | 0.87 | 1.00 | **66** | 1.6 | 0.6 | 0.86 | 0.66 | 1.11 | **182** | 4.5 | 2.0 | 0.79 | 0.68 | 0.92 |
| USA | **1.4** | **6** | 4.4 | 0.7 | 1.85 | 0.83 | 4.13 | **35** | 25.5 | 9.8 | 0.86 | 0.61 | 1.20 | **4** | 2.9 | 1.1 |  |  |  | **7** | 5.1 | 1.3 | 0.84 | 0.40 | 1.77 |
| **All North AMERICA** | **2.4** | **7** | 2.9 | 0.8 | 1.66 | 0.79 | 3.50 | **58** | 24.0 | 8.9 | 0.91 | 0.68 | 1.22 | **7** | 2.9 | 1.2 | 1.66 | 0.79 | 3.49 | **11** | 4.6 | 1.6 | 0.97 | 0.54 | 1.76 |
| Chile | **2.8** | **2** | 0.7 | 0.7 |  |  |  | **25** | 9.0 | 6.4 | 0.78 | 0.53 | 1.15 | **1** | 0.4 | 0.2 |  |  |  | **3** | 1.1 | 0.9 |  |  |  |
| **All South AMERICA** | **5.0** | **2** | 0.4 | 0.4 |  |  |  | **47** | 9.4 | 7.6 | 0.86 | 0.64 | 1.16 | **1** | 0.2 | 0.1 |  |  |  | **7** | 1.4 | 1.3 | 0.38 | 0.18 | 0.80 |
| Ethiopia | **1.2** | **0** |  |  |  |  |  | **7** | 5.7 | 5.8 | 0.98 | 0.47 | 2.06 | **0** |  |  |  |  |  | **0** |  |  |  |  |  |
| Somalia | **1.8** | **0** |  |  |  |  |  | **6** | 3.3 | 8.1 | 0.71 | 0.32 | 1.59 | **0** |  |  |  |  |  | **3** | 1.7 | 0.8 |  |  |  |
| **All AFRICA** | **7.8** | **3** | 0.4 | 0.6 |  |  |  | **41** | 5.2 | 5.3 | 0.74 | 0.55 | 1.01 | **3** | 0.4 | 0.7 |  |  |  | **13** | 1.7 | 1.2 | 0.62 | 0.36 | 1.07 |
| Iraq | **7.3** | **6** | 0.8 | 0.7 | 1.00 | 0.44 | 2.27 | **58** | 8.0 | 8.7 | 0.98 | 0.75 | 1.27 | **3** | 0.4 | 0.5 |  |  |  | **9** | 1.2 | 1.3 | 0.39 | 0.20 | 0.75 |
| Syria | **2.0** | **0** |  |  |  |  |  | **22** | 11.0 | 8.5 | 0.98 | 0.64 | 1.51 | **1** | 0.5 | 0.6 |  |  |  | **4** | 2.0 | 1.4 |  |  |  |
| Lebanon | **2.4** | **0** |  |  |  |  |  | **16** | 6.6 | 6.7 | 0.83 | 0.50 | 1.38 | **2** | 0.8 | 1.3 |  |  |  | **1** | 0.4 | 0.8 |  |  |  |
| Iran | **6.1** | **1** | 0.2 | 0.2 |  |  |  | **53** | 8.7 | 7.4 | 0.77 | 0.59 | 1.02 | **3** | 0.5 | 0.5 |  |  |  | **5** | 0.8 | 0.7 | 0.17 | 0.06 | 0.45 |
| **All Middle EAST** | **17.8** | **7** | 0.4 | 0.3 | 0.42 | 0.20 | 0.88 | **149** | 8.4 | 8.0 | 0.88 | 0.75 | 1.04 | **9** | 0.5 | 0.6 | 0.75 | 0.39 | 1.46 | **19** | 1.1 | 1.0 | 0.30 | 0.19 | 0.48 |
| India | **1.1** | **1** | 0.9 | 1.1 |  |  |  | **12** | 11.3 | 11.3 | 1.19 | 0.68 | 2.10 | **1** | 0.9 | 1.1 |  |  |  | **7** | 6.6 | 6.5 | 2.55 | 1.21 | 5.36 |
| Vietnam | **1.1** | **0** |  |  |  |  |  | **6** | 5.6 | 6.0 | 0.50 | 0.21 | 1.20 | **0** |  |  |  |  |  | **7** | 6.5 | 5.9 | 1.92 | 0.91 | 4.05 |
| Thailand | **0.5** | **0** |  |  |  |  |  | **1** | 1.9 | 0.5 |  |  |  | **1** | 1.9 | 3.3 |  |  |  | **0** |  |  |  |  |  |
| China | **0.9** | **0** |  |  |  |  |  | **6** | 6.4 | 4.8 | 0.32 | 0.13 | 0.76 | **2** | 2.1 | 1.0 |  |  |  | **2** | 2.1 | 1.5 |  |  |  |
| **All ASIA** | **8.7** | **4** | 0.5 | 0.5 |  |  |  | **58** | 6.6 | 7.5 | 0.74 | 0.57 | 0.97 | **6** | 0.7 | 0.8 | 1.08 | 0.48 | 2.41 | **23** | 2.6 | 2.9 | 0.96 | 0.64 | 1.46 |

**sTable 2** – Person years and mortality risk estimates (Crude and Age standardized mortality rates, and Hazard ratio (HRs) with 95% CI) by cancer types and immigrants’ groups, **females**, 1992 to 2016, Sweden

| Countries/region | **All site cancer** | | | | | | | **Stomach** | | | | | | **Liver** | | | | | | | **Cervical** | | | | | |
| --- | --- | --- | --- | --- | --- | --- | --- | --- | --- | --- | --- | --- | --- | --- | --- | --- | --- | --- | --- | --- | --- | --- | --- | --- | --- | --- |
|  | Person years in 100,000 | Total deaths | CMR per 100,000 | ASMR per 100,000 | Hazard ratio, 95% CI | | | Total deaths | CMR per 100,000 | ASMR per 100,000 | Hazard ratio, 95% CI | | | Total deaths | CMR per 100,000 | ASMR per 100,000 | Hazard ratio, 95% CI | | | Total deaths | | CMR per 100,000 | ASMR per 100,000 | Hazard ratio, 95% CI | | |
| **SWEDEN (ref)** | **737.7** | **229300** | 311 | 86.4 | 1.00 |  |  | 7091 | 9.6 | 2.4 | 1.00 |  |  | 5642 | 7.6 | 1.9 | 1.00 |  |  | 3213 | | 4.4 | 1.6 | 1.00 |  |  |
| **All IMMIGRANTS** | **118.1** | **25990** | 220 | 81.8 | 0.93 | 0.91 | 0.94 | 1133 | 9.6 | 3.6 | 1.39 | 1.31 | 1.49 | 740 | 6.3 | 2.2 | 1.10 | 1.01 | 1.19 | 489 | | 4.1 | 1.8 | 0.97 | 0.87 | 1.07 |
| Finland | **24.4** | **8458** | 347 | 86.4 | 0.97 | 0.95 | 0.99 | 354 | 14.5 | 3.6 | 1.43 | 1.28 | 1.59 | 227 | 9.3 | 2.2 | 1.09 | 0.96 | 1.25 | 120 | | 4.9 | 1.6 | 0.88 | 0.73 | 1.06 |
| Denmark | **4.0** | **2013** | 506 | 113 | 1.17 | 1.12 | 1.23 | 43 | 10.8 | 2.2 | 0.81 | 0.59 | 1.10 | 48 | 12.1 | 2.5 | 1.15 | 0.86 | 1.53 | 31 | | 7.8 | 2.7 | 1.23 | 0.86 | 1.77 |
| Norway | **5.2** | **2609** | 505 | 96.7 | 1.04 | 0.99 | 1.08 | 85 | 16.5 | 3.1 | 1.05 | 0.84 | 1.32 | 72 | 13.9 | 2.1 | 1.10 | 0.86 | 1.40 | 60 | | 11.6 | 3.2 | 1.63 | 1.25 | 2.13 |
| **All NORDIC** | **33.8** | **13134** | 389 | 91.3 | 1.01 | 0.99 | 1.03 | 482 | 14.3 | 3.3 | 1.26 | 1.14 | 1.39 | 348 | 10.3 | 2.2 | 1.10 | 0.98 | 1.23 | 213 | | 6.3 | 2.0 | 1.06 | 0.92 | 1.23 |
| Turkey | **3.5** | **361** | 102 | 58.1 | 0.63 | 0.57 | 0.70 | 35 | 9.9 | 5.1 | 2.13 | 1.52 | 2.98 | 11 | 3.1 | 1.5 | 0.82 | 0.45 | 1.48 | 6 | | 1.7 | 0.9 | 0.47 | 0.21 | 1.05 |
| Germany | **4.8** | **2122** | 443 | 81.7 | 0.95 | 0.91 | 0.99 | 83 | 17.3 | 3.7 | 1.26 | 1.01 | 1.58 | 45 | 9.4 | 1.5 | 0.84 | 0.62 | 1.13 | 22 | | 4.6 | 0.9 | 0.76 | 0.50 | 1.16 |
| Austria | **0.6** | **238** | 403 | 78.9 | 0.96 | 0.84 | 1.09 | 8 | 13.5 | 2.0 | 1.13 | 0.57 | 2.27 | 5 | 8.5 | 2.3 | 0.86 | 0.36 | 2.07 | 3 | | 5.1 | 0.7 |  |  |  |
| Netherlands | **0.4** | **114** | 255 | 83.5 | 0.83 | 0.69 | 1.00 | 0 |  |  |  |  |  | 1 | 2.2 | 0.5 |  |  |  | 0 | |  |  |  |  |  |
| France | **0.5** | **86** | 190 | 70 | 0.85 | 0.69 | 1.06 | 3 | 6.6 | 3.5 |  |  |  | 0 |  |  |  |  |  | 2 | | 4.4 | 1.3 |  |  |  |
| For. Yugoslavia | **6.9** | **1168** | 170 | 77.5 | 0.91 | 0.85 | 0.96 | 67 | 9.7 | 4.6 | 1.96 | 1.54 | 2.50 | 28 | 4.1 | 1.8 | 0.96 | 0.66 | 1.40 | 30 | | 4.4 | 2.0 | 1.09 | 0.76 | 1.58 |
| Bosnian-Hercegovina | **5.0** | **835** | 168 | 91.4 | 1.17 | 1.09 | 1.25 | 45 | 9.1 | 4.8 | 2.28 | 1.69 | 3.09 | 44 | 8.9 | 4.7 | 2.57 | 1.89 | 3.49 | 23 | | 4.6 | 2.6 | 1.57 | 1.03 | 2.40 |
| Italy | **0.5** | **140** | 309 | 88.6 | 0.99 | 0.83 | 1.17 | 6 | 13.2 | 3.2 | 1.53 | 0.69 | 3.42 | 7 | 15.4 | 4.1 | 1.82 | 0.82 | 4.05 | 3 | | 6.6 | 3.4 |  |  |  |
| Spain | **0.4** | **69** | 154 | 64 | 0.71 | 0.56 | 0.90 | 6 | 13.4 | 5.5 | 2.27 | 1.02 | 5.05 | 1 | 2.2 | 0.6 |  |  |  | 0 | |  |  |  |  |  |
| Greece | **0.9** | **133** | 145 | 57.5 | 0.61 | 0.52 | 0.73 | 8 | 8.7 | 3.8 | 1.21 | 0.58 | 2.54 | 3 | 3.3 | 1.4 |  |  |  | 1 | | 1.1 | 0.6 |  |  |  |
| Croatia | **0.6** | **79** | 136 | 62.6 | 0.76 | 0.61 | 0.95 | 5 | 8.6 | 3.5 | 1.85 | 0.77 | 4.46 | 2 | 3.4 | 1.7 |  |  |  | 1 | | 1.7 | 1.1 |  |  |  |
| Poland | **6.7** | **1360** | 203 | 83.2 | 0.92 | 0.87 | 0.97 | 62 | 9.3 | 3.7 | 1.50 | 1.16 | 1.94 | 37 | 5.5 | 2.3 | 1.08 | 0.77 | 1.51 | 34 | | 5.1 | 2.2 | 1.12 | 0.79 | 1.60 |
| Hungary | **1.6** | **529** | 329 | 87.6 | 1.03 | 0.94 | 1.12 | 15 | 9.3 | 2.3 | 1.07 | 0.65 | 1.78 | 19 | 11.8 | 3.0 | 1.57 | 0.99 | 2.49 | 10 | | 6.2 | 2.5 | 1.25 | 0.67 | 2.32 |
| For. Czechoslovakia | **0.8** | **309** | 365 | 83.4 | 0.99 | 0.88 | 1.10 | 13 | 15.3 | 6.3 | 1.49 | 0.86 | 2.56 | 8 | 9.4 | 1.8 | 1.13 | 0.56 | 2.26 | 3 | | 3.5 | 0.8 |  |  |  |
| Romania | **1.6** | **303** | 188 | 81.3 | 0.96 | 0.86 | 1.08 | 14 | 8.7 | 4.3 | 1.61 | 0.95 | 2.73 | 11 | 6.8 | 2.6 | 1.51 | 0.84 | 2.74 | 13 | | 8.1 | 3.9 | 2.13 | 1.23 | 3.68 |
| For. Sovjetunionen | **1.0** | **355** | 356 | 76.8 | 0.88 | 0.79 | 0.98 | 25 | 25.1 | 6.8 | 1.88 | 1.24 | 2.86 | 8 | 8.0 | 1.3 | 0.85 | 0.43 | 1.70 | 6 | | 6.0 | 1.5 | 1.03 | 0.46 | 2.30 |
| Russia | **1.2** | **100** | 86 | 64.3 | 0.74 | 0.61 | 0.90 | 11 | 9.5 | 6.3 | 3.19 | 1.76 | 5.79 | 1 | 0.9 | 0.7 |  |  |  | 5 | | 4.3 | 3.5 | 1.28 | 0.53 | 3.10 |
| Estonia | **1.3** | **817** | 610 | 82.8 | 1.08 | 1.00 | 1.16 | 45 | 33.6 | 4.8 | 1.93 | 1.44 | 2.59 | 23 | 17.2 | 2.6 | 1.14 | 0.74 | 1.74 | 20 | | 14.9 | 2.8 | 2.15 | 1.37 | 3.38 |
| UK & North Ireland | **1.0** | **227** | 217 | 86.7 | 0.97 | 0.85 | 1.10 | 6 | 5.7 | 1.9 | 0.95 | 0.43 | 2.12 | 5 | 4.8 | 1.9 | 0.98 | 0.41 | 2.37 | 6 | | 5.7 | 2.1 | 1.41 | 0.63 | 3.15 |
| **All Non-NORDIC EUROPÉ** | **43.0** | **9833** | 229 | 80.5 | 0.93 | 0.91 | 0.95 | 481 | 11.2 | 4.1 | 1.60 | 1.45 | 1.76 | 266 | 6.2 | 2.1 | 1.06 | 0.93 | 1.20 | 196 | | 4.6 | 1.9 | 1.05 | 0.90 | 1.23 |
| USA | **1.3** | **405** | 318 | 82 | 0.87 | 0.79 | 0.96 | 8 | 6.3 | 0.9 | 0.58 | 0.29 | 1.16 | 10 | 7.8 | 1.6 | 0.83 | 0.43 | 1.61 | 5 | | 3.9 | 2.2 | 0.75 | 0.31 | 1.81 |
| **All North AMERICA** | **2.3** | **492** | 216 | 75.2 | 0.82 | 0.75 | 0.90 | 14 | 6.1 | 2.1 | 0.81 | 0.48 | 1.36 | 13 | 5.7 | 2.0 | 0.88 | 0.50 | 1.55 | 8 | | 3.5 | 1.9 | 0.80 | 0.40 | 1.60 |
| Chile | **2.8** | **316** | 113 | 64.6 | 0.69 | 0.62 | 0.77 | 21 | 7.5 | 4.4 | 1.72 | 1.12 | 2.64 | 14 | 5.0 | 2.8 | 1.38 | 0.81 | 2.33 | 9 | | 3.2 | 1.7 | 0.88 | 0.46 | 1.70 |
| **All South AMERICA** | **5.3** | **507** | 95 | 62.1 | 0.67 | 0.61 | 0.73 | 29 | 5.4 | 3.5 | 1.42 | 0.98 | 2.04 | 19 | 3.6 | 2.3 | 1.13 | 0.72 | 1.77 | 13 | | 2.4 | 1.4 | 0.71 | 0.41 | 1.23 |
| Ethiopia | **1.1** | **63** | 58 | 70 | 0.91 | 0.71 | 1.17 | 1 | 0.9 | 2.4 |  |  |  | 5 | 4.6 | 9.9 | 3.56 | 1.48 | 8.57 | 2 | | 1.8 | 2.4 |  |  |  |
| Somalia | **1.8** | **62** | 35 | 42.3 | 0.51 | 0.39 | 0.65 | 1 | 0.6 | 0.8 |  |  |  | 8 | 4.6 | 7.7 | 2.66 | 1.32 | 5.36 | 1 | | 0.6 | 1.4 |  |  |  |
| **All AFRICA** | **6.5** | **316** | 49 | 53 | 0.66 | 0.59 | 0.73 | 9 | 1.4 | 1.5 | 0.69 | 0.36 | 1.33 | 19 | 2.9 | 4.1 | 1.71 | 1.07 | 2.73 | 11 | | 1.7 | 1.4 | 0.60 | 0.32 | 1.12 |
| Iraq | **5.6** | **342** | 61 | 55.1 | 0.63 | 0.57 | 0.70 | 11 | 2.0 | 1.8 | 0.79 | 0.44 | 1.43 | 9 | 1.6 | 1.5 | 0.75 | 0.39 | 1.46 | 2 | | 0.4 | 0.4 |  |  |  |
| Syria | **1.9** | **147** | 79 | 58.9 | 0.70 | 0.59 | 0.82 | 13 | 7.0 | 5.1 | 2.30 | 1.33 | 3.98 | 5 | 2.7 | 2.7 | 1.05 | 0.44 | 2.52 | 1 | | 0.5 | 0.3 |  |  |  |
| Lebanon | **1.9** | **91** | 48 | 50.9 | 0.52 | 0.42 | 0.65 | 6 | 3.2 | 4.1 | 1.32 | 0.59 | 2.95 | 1 | 0.5 | 0.6 |  |  |  | 1 | | 0.5 | 0.4 |  |  |  |
| Iran | **5.2** | **369** | 71 | 47.8 | 0.55 | 0.50 | 0.61 | 30 | 5.8 | 3.6 | 1.63 | 1.14 | 2.35 | 7 | 1.4 | 0.9 | 0.47 | 0.22 | 0.99 | 5 | | 1.0 | 0.6 | 0.30 | 0.12 | 0.72 |
| **All Middle EAST** | **14.5** | **949** | 65 | 52.3 | 0.60 | 0.56 | 0.64 | 60 | 4.1 | 3.3 | 1.39 | 1.07 | 1.80 | 22 | 1.5 | 1.3 | 0.61 | 0.40 | 0.93 | 9 | | 0.6 | 0.5 | 0.21 | 0.11 | 0.40 |
| India | **1.2** | **58** | 48 | 57.2 | 0.63 | 0.48 | 0.81 | 3 | 2.5 | 2.8 |  |  |  | 3 | 2.5 | 3.5 |  |  |  | 4 | | 3.3 | 4.7 |  |  |  |
| Vietnam | **1.2** | **75** | 62 | 53.6 | 0.55 | 0.44 | 0.69 | 8 | 6.6 | 4.7 | 2.12 | 1.06 | 4.25 | 6 | 4.9 | 4.3 | 1.92 | 0.86 | 4.29 | 5 | | 4.1 | 2.5 | 1.24 | 0.51 | 2.99 |
| Thailand | **2.7** | **121** | 45 | 50.8 | 0.62 | 0.52 | 0.74 | 11 | 4.1 | 2.4 | 2.27 | 1.25 | 4.13 | 9 | 3.3 | 5.6 | 2.49 | 1.29 | 4.81 | 5 | | 1.8 | 2.0 | 0.52 | 0.22 | 1.26 |
| China | **1.3** | **123** | 98 | 64.3 | 0.65 | 0.54 | 0.77 | 7 | 5.6 | 3.7 | 1.31 | 0.63 | 2.76 | 10 | 7.9 | 5.6 | 2.13 | 1.11 | 4.11 | 7 | | 5.6 | 3.6 | 1.54 | 0.73 | 3.24 |
| **All ASIA** | **12.5** | **738** | 59 | 61.8 | 0.65 | 0.60 | 0.70 | 57 | 4.5 | 3.8 | 1.86 | 1.43 | 2.43 | 52 | 4.1 | 4.8 | 2.06 | 1.55 | 2.74 | 38 | | 3.0 | 2.6 | 1.04 | 0.75 | 1.45 |

…continued (sTable2)

| **Countries/region** | **Lung** | | | | | | | **Esophageal** | | | | | | **Laryngeal** | | | | | | **Kidney** | | | | | |
| --- | --- | --- | --- | --- | --- | --- | --- | --- | --- | --- | --- | --- | --- | --- | --- | --- | --- | --- | --- | --- | --- | --- | --- | --- | --- |
|  | Person years in 100,000 | Total deaths | CMR per 100,000 | ASMR per 100,000 | Hazard ratio, 95% CI | | | Total deaths | CMR per 100,000 | ASMR per 100,000 | Hazard ratio, 95% CI | | | Total deaths | CMR per 100,000 | ASMR per 100,000 | Hazard ratio, 95% CI | | | Total deaths | CMR per 100,000 | ASMR per 100,000 | Hazard ratio, 95% CI | | |
| **SWEDEN (ref)** | **737.7** | **30,688** | 41.6 | 13.5 | 1.00 |  |  | **2,372** | 3.2 | 0.8 | 1.00 |  |  | **196** | 0.3 | 0.1 | 1.00 |  |  | **6081** | 8.2 | 2.1 | 1.00 |  |  |
| **All IMMIGRANTS** | **118.1** | **4,258** | 36.1 | 13.8 | 0.99 | 0.96 | 1.03 | **311** | 2.6 | 0.9 | 1.07 | 0.94 | 1.21 | **34** | 0.3 | 0.1 | 1.23 | 0.84 | 1.81 | **631** | 5.3 | 1.9 | 0.92 | 0.85 | 1.01 |
| Finland | **24.4** | **1,465** | 60.2 | 15.3 | 1.02 | 0.97 | 1.08 | **117** | 4.8 | 1.1 | 1.23 | 1.02 | 1.49 | **11** | 0.5 | 0.1 | 1.13 | 0.60 | 2.15 | **252** | 10.3 | 2.3 | 1.10 | 0.97 | 1.25 |
| Denmark | **4.0** | **482** | 121.2 | 27.5 | 1.93 | 1.77 | 2.12 | **22** | 5.5 | 1.2 | 1.20 | 0.78 | 1.84 | **1** | 0.3 | 0.1 | 0.61 | 0.09 | 4.38 | **52** | 13.1 | 3.0 | 1.12 | 0.84 | 1.48 |
| Norway | **5.2** | **455** | 88.1 | 19.3 | 1.30 | 1.18 | 1.43 | **34** | 6.6 | 1.0 | 1.20 | 0.84 | 1.70 | **6** | 1.2 | 0.4 | 2.46 | 1.09 | 5.57 | **65** | 12.6 | 2.5 | 0.95 | 0.74 | 1.22 |
| **All NORDIC** | **33.8** | **2,416** | 71.5 | 17.4 | 1.18 | 1.14 | 1.24 | **173** | 5.1 | 1.1 | 1.22 | 1.04 | 1.43 | **18** | 0.5 | 0.1 | 1.31 | 0.79 | 2.16 | **365** | 10.8 | 2.4 | 1.07 | 0.96 | 1.19 |
| Turkey | **3.5** | **46** | 13.0 | 8.3 | 0.53 | 0.39 | 0.71 | **1** | 0.3 | 0.2 |  |  |  | **1** | 0.3 | 0.2 |  |  |  | **8** | 2.3 | 1.1 | 0.64 | 0.32 | 1.29 |
| Germany | **4.8** | **327** | 68.3 | 13.5 | 1.02 | 0.92 | 1.14 | **32** | 6.7 | 1.1 | 1.40 | 0.99 | 1.98 | **2** | 0.4 | 0.1 |  |  |  | **60** | 12.5 | 1.7 | 1.05 | 0.81 | 1.35 |
| Austria | **0.6** | **23** | 39.0 | 6.4 | 0.67 | 0.44 | 1.01 | **2** | 3.4 | 0.9 |  |  |  | **0** |  |  |  |  |  | **4** | 6.8 | 1.8 |  |  |  |
| Netherlands | **0.4** | **18** | 40.3 | 12.1 | 0.95 | 0.60 | 1.51 | **1** | 2.2 | 0.5 |  |  |  | **0** |  |  |  |  |  | **4** | 9.0 | 3.8 |  |  |  |
| France | **0.5** | **12** | 26.4 | 9.3 | 0.81 | 0.45 | 1.47 | **0** |  |  |  |  |  | **1** | 2.2 | 0.6 |  |  |  | **1** | 2.2 | 1.5 |  |  |  |
| For. Yugoslavia | **6.9** | **190** | 27.6 | 12.6 | 0.83 | 0.72 | 0.96 | **7** | 1.0 | 0.5 | 0.54 | 0.26 | 1.14 | **2** | 0.3 | 0.1 |  |  |  | **24** | 3.5 | 1.5 | 0.84 | 0.56 | 1.25 |
| Bosnian-Hercegovina | **5.0** | **127** | 25.5 | 13.9 | 1.11 | 0.93 | 1.32 | **7** | 1.4 | 0.7 | 0.96 | 0.45 | 2.05 | **2** | 0.4 | 0.2 |  |  |  | **13** | 2.6 | 1.5 | 0.90 | 0.52 | 1.56 |
| Italy | **0.5** | **28** | 61.7 | 16.9 | 1.30 | 0.88 | 1.90 | **2** | 4.4 | 1.9 |  |  |  | **1** | 2.2 | 1.1 |  |  |  | **4** | 8.8 | 2.1 |  |  |  |
| Spain | **0.4** | **8** | 17.8 | 6.9 | 0.53 | 0.27 | 1.06 | **1** | 2.2 | 0.7 |  |  |  | **0** |  |  |  |  |  | **2** | 4.5 | 1.5 |  |  |  |
| Greece | **0.9** | **8** | 8.7 | 3.1 | 0.24 | 0.12 | 0.47 | **0** |  |  |  |  |  | **0** |  |  |  |  |  | **4** | 4.3 | 1.7 |  |  |  |
| Croatia | **0.6** | **11** | 19.0 | 8.6 | 0.60 | 0.33 | 1.08 | **2** | 3.4 | 1.5 |  |  |  | **0** |  |  |  |  |  | **1** | 1.7 | 0.7 |  |  |  |
| Poland | **6.7** | **257** | 38.3 | 15.6 | 1.08 | 0.96 | 1.23 | **12** | 1.8 | 0.7 | 0.81 | 0.46 | 1.44 | **3** | 0.4 | 0.2 |  |  |  | **25** | 3.7 | 1.7 | 0.74 | 0.49 | 1.10 |
| Hungary | **1.6** | **99** | 61.7 | 16.9 | 1.24 | 1.01 | 1.51 | **3** | 1.9 | 0.4 |  |  |  | **0** |  |  |  |  |  | **12** | 7.5 | 2.1 | 0.96 | 0.55 | 1.70 |
| For. Czechoslovakia | **0.8** | **39** | 46.0 | 10.4 | 0.90 | 0.66 | 1.23 | **1** | 1.2 | 0.1 |  |  |  | **1** | 1.2 | 0.3 |  |  |  | **7** | 8.3 | 1.7 | 0.90 | 0.43 | 1.88 |
| Romania | **1.6** | **49** | 30.5 | 12.6 | 1.02 | 0.77 | 1.36 | **2** | 1.2 | 0.5 |  |  |  | **1** | 0.6 | 0.4 |  |  |  | **7** | 4.4 | 1.7 | 1.00 | 0.48 | 2.10 |
| For. Sovjetunionen | **1.0** | **31** | 31.1 | 7.0 | 0.57 | 0.39 | 0.81 | **2** | 2.0 | 0.2 |  |  |  | **0** |  |  |  |  |  | **10** | 10.0 | 2.3 | 0.93 | 0.48 | 1.78 |
| Russia | **1.2** | **7** | 6.0 | 5.7 | 0.34 | 0.16 | 0.72 | **0** |  |  |  |  |  | **0** |  |  |  |  |  | **1** | 0.9 | 0.5 |  |  |  |
| Estonia | **1.3** | **93** | 69.4 | 12.1 | 1.02 | 0.83 | 1.26 | **9** | 6.7 | 1.3 | 1.03 | 0.51 | 2.06 | **0** |  |  |  |  |  | **17** | 12.7 | 2.0 | 0.86 | 0.53 | 1.38 |
| UK & North Ireland | **1.0** | **41** | 39.3 | 15.4 | 1.20 | 0.88 | 1.63 | **5** | 4.8 | 1.9 | 2.22 | 0.92 | 5.34 | **0** |  |  |  |  |  | **3** | 2.9 | 1.3 |  |  |  |
| **All Non-NORDIC EUROPÉ** | **43.0** | **1,475** | 34.3 | 12.4 | 0.92 | 0.87 | 0.97 | **92** | 2.1 | 0.7 | 0.85 | 0.69 | 1.06 | **14** | 0.3 | 0.2 | 1.41 | 0.80 | 2.47 | **218** | 5.1 | 1.7 | 0.88 | 0.76 | 1.01 |
| USA | **1.3** | **59** | 46.3 | 12.4 | 1.06 | 0.81 | 1.38 | **7** | 5.5 | 1.9 | 1.56 | 0.74 | 3.27 | **0** |  |  |  |  |  | **15** | 11.8 | 2.5 | 1.16 | 0.67 | 1.99 |
| **All North AMERICA** | **2.3** | **69** | 30.3 | 10.2 | 0.90 | 0.70 | 1.15 | **8** | 3.5 | 1.3 | 1.39 | 0.69 | 2.79 | **0** |  |  |  |  |  | **16** | 7.0 | 2.2 | 1.01 | 0.60 | 1.70 |
| Chile | **2.8** | **41** | 14.7 | 9.0 | 0.53 | 0.39 | 0.72 | **5** | 1.8 | 0.9 | 1.10 | 0.46 | 2.65 | **0** |  |  |  |  |  | **7** | 2.5 | 1.6 | 0.70 | 0.34 | 1.48 |
| **All South AMERICA** | **5.3** | **69** | 12.9 | 9.1 | 0.55 | 0.43 | 0.70 | **9** | 1.7 | 1.1 | 1.21 | 0.63 | 2.33 | **0** |  |  |  |  |  | **13** | 2.4 | 1.7 | 0.82 | 0.47 | 1.41 |
| Ethiopia | **1.1** | **4** | 3.7 | 2.1 |  |  |  | **0** | 0.0 |  |  |  |  | **0** |  |  |  |  |  | **1** | 0.9 | 2.0 |  |  |  |
| Somalia | **1.8** | **6** | 3.4 | 4.9 | 0.33 | 0.15 | 0.73 | **6** | 3.4 | 3.0 | 5.18 | 2.29 | 11.72 | **0** |  |  |  |  |  | **0** |  |  |  |  |  |
| **All AFRICA** | **6.5** | **33** | 5.1 | 6.4 | 0.43 | 0.30 | 0.61 | **9** | 1.4 | 1.6 | 2.16 | 1.11 | 4.20 | **0** |  |  |  |  |  | **2** | 0.3 | 0.4 |  |  |  |
| Iraq | **5.6** | **29** | 5.2 | 5.1 | 0.34 | 0.24 | 0.50 | **2** | 0.4 | 0.4 |  |  |  | **1** | 0.2 | 0.1 |  |  |  | **1** | 0.2 | 0.2 |  |  |  |
| Syria | **1.9** | **13** | 7.0 | 4.6 | 0.38 | 0.22 | 0.66 | **0** |  |  |  |  |  | **0** |  |  |  |  |  | **1** | 0.5 | 0.3 |  |  |  |
| Lebanon | **1.9** | **22** | 11.7 | 12.7 | 0.77 | 0.50 | 1.18 | **0** |  |  |  |  |  | **1** | 0.5 | 0.4 |  |  |  | **1** | 0.5 | 0.8 |  |  |  |
| Iran | **5.2** | **35** | 6.8 | 4.4 | 0.33 | 0.23 | 0.45 | **8** | 1.5 | 1.2 | 1.26 | 0.63 | 2.53 | **0** |  |  |  |  |  | **3** | 0.6 | 0.4 |  |  |  |
| **All Middle EAST** | **14.5** | **99** | 6.8 | 5.4 | 0.39 | 0.32 | 0.47 | **10** | 0.7 | 0.6 | 0.68 | 0.37 | 1.28 | **2** | 0.1 | 0.1 |  |  |  | **6** | 0.4 | 0.4 | 0.20 | 0.09 | 0.44 |
| India | **1.2** | **4** | 3.3 | 4.0 |  |  |  | **0** |  |  |  |  |  | **0** |  |  |  |  |  | **1** | 0.8 | 0.7 |  |  |  |
| Vietnam | **1.2** | **9** | 7.4 | 6.6 | 0.39 | 0.20 | 0.75 | **0** |  |  |  |  |  | **0** |  |  |  |  |  | **1** | 0.8 | 0.7 |  |  |  |
| Thailand | **2.7** | **21** | 7.7 | 9.3 | 0.64 | 0.42 | 0.98 | **2** | 0.7 | 0.9 |  |  |  | **0** |  |  |  |  |  | **0** |  |  |  |  |  |
| China | **1.3** | **18** | 14.3 | 9.0 | 0.70 | 0.44 | 1.13 | **1** | 0.8 | 0.4 |  |  |  | **0** |  |  |  |  |  | **0** |  |  |  |  |  |
| **All ASIA** | **12.5** | **93** | 7.4 | 8.1 | 0.54 | 0.44 | 0.66 | **10** | 0.8 | 1.1 | 1.02 | 0.55 | 1.91 | **0** |  |  |  |  |  | **7** | 0.6 | 0.7 | 0.33 | 0.16 | 0.70 |

…continued (sTable2)

| **Countries/region** | **Bladder** | | | | | | | **Colorectal** | | | | | | **Pancreas** | | | | | | **Breast** | | | | | |
| --- | --- | --- | --- | --- | --- | --- | --- | --- | --- | --- | --- | --- | --- | --- | --- | --- | --- | --- | --- | --- | --- | --- | --- | --- | --- |
|  | Person years in 100,000 | Total deaths | CMR per 100,000 | ASMR per 100,000 | Hazard ratio, 95% CI | | | Total deaths | CMR per 100,000 | ASMR per 100,000 | Hazard ratio, 95% CI | | | Total deaths | CMR per 100,000 | ASMR per 100,000 | Hazard ratio, 95% CI | | | Total deaths | CMR per 100,000 | ASMR per 100,000 | Hazard ratio, 95% CI | | |
| **SWEDEN (ref)** | **737.7** | **4,288** | 5.8 | 1.2 | 1.00 |  |  | **28,663** | 38.9 | 9.4 | 1.00 |  |  | **17,947** | 24.3 | 6.3 | 1.00 |  |  | **32,613** | 44.2 | 14.5 | 1.00 |  |  |
| **All IMMIGRANTS** | **118.1** | **426** | 3.6 | 1.0 | 0.91 | 0.82 | 1.01 | **2,857** | 24.2 | 8.3 | 0.88 | 0.85 | 0.92 | **1,935** | 16.4 | 5.7 | 0.91 | 0.87 | 0.96 | **3,755** | 31.8 | 13.3 | 0.90 | 0.87 | 0.93 |
| Finland | **24.4** | **117** | 4.8 | 1.0 | 0.80 | 0.66 | 0.96 | **802** | 32.9 | 7.6 | 0.78 | 0.73 | 0.84 | **724** | 29.7 | 6.8 | 1.06 | 0.98 | 1.14 | **1,164** | 47.8 | 13.5 | 0.94 | 0.89 | 1.00 |
| Denmark | **4.0** | **41** | 10.3 | 1.7 | 1.26 | 0.91 | 1.73 | **262** | 65.9 | 12.4 | 1.25 | 1.11 | 1.42 | **128** | 32.2 | 7.1 | 0.96 | 0.81 | 1.15 | **240** | 60.4 | 16.1 | 1.01 | 0.89 | 1.16 |
| Norway | **5.2** | **61** | 11.8 | 1.3 | 1.21 | 0.93 | 1.57 | **349** | 67.6 | 11.5 | 1.11 | 1.00 | 1.23 | **188** | 36.4 | 6.2 | 0.94 | 0.81 | 1.09 | **269** | 52.1 | 13.1 | 0.80 | 0.70 | 0.90 |
| **All NORDIC** | **33.8** | **219** | 6.5 | 1.1 | 0.95 | 0.82 | 1.09 | **1,418** | 42.0 | 8.8 | 0.91 | 0.87 | 0.97 | **1,042** | 30.9 | 6.8 | 1.02 | 0.96 | 1.09 | **1,680** | 49.7 | 13.7 | 0.92 | 0.88 | 0.97 |
| Turkey | **3.5** | **3** | 0.8 | 0.5 |  |  |  | **34** | 9.6 | 5.1 | 0.54 | 0.38 | 0.75 | **25** | 7.1 | 3.8 | 0.63 | 0.42 | 0.93 | **43** | 12.2 | 6.9 | 0.49 | 0.36 | 0.66 |
| Germany | **4.8** | **47** | 9.8 | 1.1 | 1.13 | 0.84 | 1.52 | **284** | 59.3 | 9.8 | 1.03 | 0.92 | 1.16 | **170** | 35.5 | 5.6 | 0.96 | 0.82 | 1.11 | **288** | 60.2 | 15.1 | 0.93 | 0.82 | 1.05 |
| Austria | **0.6** | **8** | 13.5 | 1.7 | 1.77 | 0.88 | 3.54 | **32** | 54.2 | 9.5 | 1.02 | 0.72 | 1.45 | **20** | 33.9 | 6.9 | 0.96 | 0.62 | 1.51 | **44** | 74.5 | 15.3 | 1.29 | 0.96 | 1.74 |
| Netherlands | **0.4** | **3** | 6.7 | 2.3 |  |  |  | **11** | 24.6 | 6.2 | 0.58 | 0.30 | 1.11 | **11** | 24.6 | 7.3 | 1.05 | 0.58 | 1.90 | **20** | 44.8 | 19.4 | 0.88 | 0.56 | 1.40 |
| France | **0.5** | **3** | 6.6 | 2.3 |  |  |  | **13** | 28.6 | 9.7 | 1.12 | 0.65 | 1.93 | **7** | 15.4 | 4.5 | 0.92 | 0.44 | 1.94 | **16** | 35.3 | 15.0 | 1.03 | 0.63 | 1.68 |
| For. Yugoslavia | **6.9** | **17** | 2.5 | 1.0 | 0.91 | 0.56 | 1.47 | **98** | 14.2 | 6.2 | 0.68 | 0.56 | 0.84 | **78** | 11.3 | 5.1 | 0.82 | 0.66 | 1.03 | **211** | 30.6 | 14.5 | 1.09 | 0.95 | 1.25 |
| Bosnian-Hercegovina | **5.0** | **13** | 2.6 | 1.1 | 1.25 | 0.72 | 2.17 | **70** | 14.1 | 7.7 | 0.92 | 0.72 | 1.16 | **47** | 9.5 | 5.4 | 0.89 | 0.66 | 1.19 | **126** | 25.3 | 14.4 | 1.15 | 0.96 | 1.38 |
| Italy | **0.5** | **1** | 2.2 | 0.4 |  |  |  | **18** | 39.7 | 13.5 | 1.04 | 0.65 | 1.68 | **5** | 11.0 | 1.8 | 0.47 | 0.19 | 1.12 | **15** | 33.1 | 10.1 | 0.78 | 0.47 | 1.30 |
| Spain | **0.4** | **2** | 4.5 | 1.6 |  |  |  | **6** | 13.4 | 5.4 | 0.55 | 0.25 | 1.23 | **8** | 17.8 | 7.0 | 1.10 | 0.55 | 2.19 | **12** | 26.8 | 12.1 | 0.81 | 0.46 | 1.42 |
| Greece | **0.9** | **3** | 3.3 | 1.2 |  |  |  | **18** | 19.6 | 7.4 | 0.80 | 0.50 | 1.27 | **5** | 5.4 | 2.0 | 0.33 | 0.14 | 0.78 | **32** | 34.8 | 15.0 | 0.92 | 0.65 | 1.32 |
| Croatia | **0.6** | **1** | 1.7 | 0.7 |  |  |  | **9** | 15.5 | 8.1 | 0.81 | 0.42 | 1.55 | **4** | 6.9 | 3.2 |  |  |  | **11** | 19.0 | 9.0 | 0.69 | 0.38 | 1.24 |
| Poland | **6.7** | **27** | 4.0 | 1.3 | 1.19 | 0.82 | 1.75 | **140** | 20.9 | 8.1 | 0.85 | 0.72 | 1.01 | **97** | 14.5 | 5.7 | 0.86 | 0.70 | 1.05 | **204** | 30.4 | 13.1 | 0.89 | 0.77 | 1.02 |
| Hungary | **1.6** | **8** | 5.0 | 0.9 | 0.95 | 0.48 | 1.91 | **63** | 39.2 | 10.2 | 1.05 | 0.82 | 1.35 | **49** | 30.5 | 7.7 | 1.20 | 0.91 | 1.60 | **76** | 47.3 | 13.7 | 1.00 | 0.80 | 1.26 |
| For. Czechoslovakia | **0.8** | **5** | 5.9 | 1.0 | 0.93 | 0.39 | 2.23 | **38** | 44.8 | 8.3 | 1.02 | 0.74 | 1.40 | **30** | 35.4 | 6.8 | 1.18 | 0.82 | 1.69 | **40** | 47.2 | 12.1 | 0.85 | 0.62 | 1.16 |
| Romania | **1.6** | **7** | 4.4 | 1.6 | 1.38 | 0.66 | 2.91 | **33** | 20.5 | 7.2 | 0.94 | 0.67 | 1.32 | **18** | 11.2 | 4.9 | 0.75 | 0.48 | 1.20 | **58** | 36.1 | 17.1 | 1.20 | 0.93 | 1.56 |
| For. Sovjetunionen | **1.0** | **5** | 5.0 | 0.6 | 0.67 | 0.28 | 1.62 | **53** | 53.1 | 9.2 | 1.08 | 0.82 | 1.42 | **30** | 30.1 | 6.1 | 0.97 | 0.67 | 1.39 | **41** | 41.1 | 12.6 | 0.69 | 0.50 | 0.96 |
| Russia | **1.2** | **1** | 0.9 | 1.0 |  |  |  | **18** | 15.5 | 10.2 | 1.32 | 0.83 | 2.10 | **3** | 2.6 | 2.1 |  |  |  | **17** | 14.7 | 12.6 | 0.68 | 0.42 | 1.10 |
| Estonia | **1.3** | **20** | 14.9 | 1.2 | 1.22 | 0.78 | 1.92 | **117** | 87.3 | 8.8 | 1.16 | 0.97 | 1.40 | **76** | 56.7 | 7.1 | 1.25 | 0.99 | 1.57 | **89** | 66.4 | 11.0 | 0.89 | 0.72 | 1.10 |
| UK & North Ireland | **1.0** | **3** | 2.9 | 1.2 |  |  |  | **29** | 27.8 | 11.2 | 1.10 | 0.76 | 1.58 | **14** | 13.4 | 5.4 | 0.74 | 0.43 | 1.28 | **41** | 39.3 | 16.3 | 1.05 | 0.77 | 1.44 |
| **All Non-NORDIC EUROPÉ** | **43.0** | **181** | 4.2 | 1.1 | 1.03 | 0.88 | 1.20 | **1,141** | 26.5 | 8.4 | 0.93 | 0.87 | 0.99 | **728** | 16.9 | 5.5 | 0.90 | 0.84 | 0.97 | **1,469** | 34.2 | 12.1 | 0.93 | 0.89 | 0.99 |
| USA | **1.3** | **7** | 5.5 | 0.4 | 0.81 | 0.38 | 1.69 | **50** | 39.2 | 9.6 | 0.88 | 0.66 | 1.16 | **26** | 20.4 | 4.8 | 0.74 | 0.50 | 1.09 | **69** | 54.1 | 15.7 | 1.00 | 0.78 | 1.27 |
| **All North AMERICA** | **2.3** | **9** | 4.0 | 0.9 | 0.85 | 0.44 | 1.64 | **56** | 24.6 | 7.8 | 0.78 | 0.59 | 1.01 | **32** | 14.1 | 4.8 | 0.72 | 0.51 | 1.03 | **87** | 38.2 | 14.4 | 0.95 | 0.76 | 1.18 |
| Chile | **2.8** | **3** | 1.1 | 0.6 |  |  |  | **35** | 12.5 | 7.0 | 0.72 | 0.52 | 1.01 | **25** | 9.0 | 5.1 | 0.77 | 0.52 | 1.14 | **40** | 14.3 | 7.7 | 0.54 | 0.39 | 0.74 |
| **All South AMERICA** | **5.3** | **5** | 0.9 | 0.5 | 0.46 | 0.19 | 1.11 | **55** | 10.3 | 6.7 | 0.69 | 0.53 | 0.90 | **36** | 6.7 | 4.6 | 0.68 | 0.49 | 0.94 | **83** | 15.6 | 9.7 | 0.65 | 0.53 | 0.81 |
| Ethiopia | **1.1** | **0** |  |  |  |  |  | **6** | 5.5 | 2.4 | 0.89 | 0.40 | 1.98 | **2** | 1.8 | 4.8 |  |  |  | **16** | 14.7 | 9.0 | 1.29 | 0.79 | 2.11 |
| Somalia | **1.8** | **1** | 0.6 | 0.6 |  |  |  | **3** | 1.7 | 1.3 |  |  |  | **1** | 0.6 | 0.6 |  |  |  | **7** | 4.0 | 3.6 | 0.38 | 0.18 | 0.79 |
| **All AFRICA** | **6.5** | **1** | 0.2 | 0.2 |  |  |  | **23** | 3.6 | 2.8 | 0.49 | 0.33 | 0.74 | **15** | 2.3 | 3.2 | 0.49 | 0.30 | 0.82 | **72** | 11.2 | 9.3 | 0.86 | 0.68 | 1.09 |
| Iraq | **5.6** | **3** | 0.5 | 0.5 |  |  |  | **38** | 6.8 | 6.2 | 0.71 | 0.52 | 0.98 | **20** | 3.6 | 3.3 | 0.56 | 0.36 | 0.87 | **108** | 19.3 | 16.0 | 1.15 | 0.94 | 1.39 |
| Syria | **1.9** | **0** |  |  |  |  |  | **13** | 7.0 | 5.2 | 0.60 | 0.35 | 1.04 | **10** | 5.4 | 4.5 | 0.70 | 0.38 | 1.30 | **39** | 20.9 | 13.7 | 1.12 | 0.81 | 1.53 |
| Lebanon | **1.9** | **2** | 1.1 | 1.0 |  |  |  | **8** | 4.3 | 4.9 | 0.49 | 0.25 | 0.98 | **1** | 0.5 | 0.6 |  |  |  | **19** | 10.1 | 8.8 | 0.61 | 0.39 | 0.97 |
| Iran | **5.2** | **4** | 0.8 | 0.6 |  |  |  | **33** | 6.4 | 4.3 | 0.45 | 0.32 | 0.64 | **17** | 3.3 | 2.2 | 0.36 | 0.22 | 0.58 | **79** | 15.3 | 9.5 | 0.71 | 0.57 | 0.88 |
| **All Middle EAST** | **14.5** | **9** | 0.6 | 0.5 | 0.43 | 0.22 | 0.82 | **92** | 6.3 | 5.2 | 0.57 | 0.46 | 0.70 | **48** | 3.3 | 2.7 | 0.45 | 0.34 | 0.60 | **245** | 16.9 | 12.1 | 0.90 | 0.79 | 1.03 |
| India | **1.2** | **0** |  |  |  |  |  | **6** | 4.9 | 5.1 | 0.63 | 0.28 | 1.40 | **2** | 1.6 | 2.1 |  |  |  | **15** | 12.3 | 13.5 | 0.94 | 0.56 | 1.55 |
| Vietnam | **1.2** | **0** |  |  |  |  |  | **9** | 7.4 | 7.6 | 0.64 | 0.33 | 1.23 | **6** | 4.9 | 5.0 | 0.64 | 0.29 | 1.43 | **8** | 6.6 | 5.4 | 0.36 | 0.18 | 0.72 |
| Thailand | **2.7** | **1** | 0.4 | 3.0 |  |  |  | **5** | 1.8 | 4.6 | 0.30 | 0.13 | 0.73 | **3** | 1.1 | 0.7 |  |  |  | **23** | 8.5 | 7.6 | 0.55 | 0.36 | 0.83 |
| China | **1.3** | **0** |  |  |  |  |  | **19** | 15.1 | 8.0 | 0.89 | 0.56 | 1.41 | **4** | 3.2 | 3.3 |  |  |  | **13** | 10.3 | 6.9 | 0.43 | 0.25 | 0.74 |
| **All ASIA** | **12.5** | **2** | 0.2 | 0.2 |  |  |  | **69** | 5.5 | 6.1 | 0.61 | 0.48 | 0.78 | **34** | 2.7 | 3.5 | 0.47 | 0.34 | 0.66 | **116** | 9.3 | 8.5 | 0.55 | 0.45 | 0.66 |

…continued (sTable2)

| Countries/region | **Corpus-uteri** | | | | | | | **Ovarian** | | | | | | **Vulva & vagina** | | | | | | **Brain and CNS** | | | | | |
| --- | --- | --- | --- | --- | --- | --- | --- | --- | --- | --- | --- | --- | --- | --- | --- | --- | --- | --- | --- | --- | --- | --- | --- | --- | --- |
|  | Person years in 100,000 | Total deaths | CMR per 100,000 | ASMR per 100,000 | Hazard ratio, 95% CI | | | Total deaths | CMR per 100,000 | ASMR per 100,000 | Hazard ratio, 95% CI | | | Total deaths | CMR per 100,000 | ASMR per 100,000 | Hazard ratio, 95% CI | | | Total deaths | CMR per 100,000 | ASMR per 100,000 | Hazard ratio, 95% CI | | |
| **SWEDEN (ref)** | **737.7** | **7,218** | 9.8 | 2.5 | 1.00 |  |  | **13,339** | 18.1 | 6.1 | 1.00 |  |  | **1,834** | 2.5 | 0.5 | 1.00 |  |  | **6,484** | 8.8 | 3.3 | 1.00 |  |  |
| **All IMMIGRANTS** | **118.1** | **749** | 6.3 | 2.2 | 0.88 | 0.81 | 0.95 | **1,543** | 13.1 | 5.3 | 0.91 | 0.86 | 0.96 | **157** | 1.3 | 0.4 | 0.80 | 0.68 | 0.95 | **721** | 6.1 | 2.6 | 0.82 | 0.76 | 0.89 |
| Finland | **24.4** | **243** | 10.0 | 2.2 | 0.88 | 0.78 | 1.01 | **517** | 21.2 | 5.6 | 0.96 | 0.87 | 1.04 | **51** | 2.1 | 0.5 | 0.85 | 0.64 | 1.20 | **236** | 9.7 | 2.7 | 0.89 | 0.78 | 1.02 |
| Denmark | **4.0** | **32** | 8.0 | 1.9 | 0.59 | 0.41 | 0.84 | **122** | 30.7 | 8.1 | 1.19 | 0.99 | 1.43 | **7** | 1.8 | 0.3 | 0.56 | 0.27 | 1.18 | **45** | 11.3 | 3.5 | 0.95 | 0.71 | 1.28 |
| Norway | **5.2** | **73** | 14.1 | 2.6 | 0.92 | 0.73 | 1.16 | **147** | 28.5 | 6.5 | 1.05 | 0.89 | 1.24 | **27** | 5.2 | 0.8 | 1.38 | 0.95 | 2.03 | **59** | 11.4 | 2.7 | 0.94 | 0.73 | 1.22 |
| **All NORDIC** | **33.8** | **351** | 10.4 | 2.2 | 0.86 | 0.77 | 0.96 | **792** | 23.4 | 6.0 | 1.01 | 0.93 | 1.08 | **85** | 2.5 | 0.5 | 0.92 | 0.74 | 1.15 | **342** | 10.1 | 2.9 | 0.91 | 0.81 | 1.01 |
| Turkey | **3.5** | **12** | 3.4 | 1.9 | 0.70 | 0.39 | 1.26 | **19** | 5.4 | 3.3 | 0.54 | 0.34 | 0.86 | **3** | 0.8 | 0.5 |  |  |  | **15** | 4.2 | 2.8 | 0.73 | 0.43 | 1.24 |
| Germany | **4.8** | **60** | 12.5 | 1.8 | 0.85 | 0.66 | 1.09 | **141** | 29.5 | 6.3 | 1.03 | 0.87 | 1.22 | **12** | 2.5 | 0.4 | 0.75 | 0.42 | 1.32 | **46** | 9.6 | 2.2 | 0.74 | 0.55 | 0.99 |
| Austria | **0.6** | **9** | 15.2 | 2.6 | 1.02 | 0.51 | 2.05 | **17** | 28.8 | 7.9 | 1.19 | 0.74 | 1.91 | **1** | 1.7 | 0.2 |  |  |  | **3** | 5.1 | 2.7 |  |  |  |
| Netherlands | **0.4** | **5** | 11.2 | 4.0 | 1.19 | 0.50 | 2.87 | **11** | 24.6 | 9.1 | 1.31 | 0.72 | 2.36 | **0** |  |  |  |  |  | **1** | 2.2 | 0.5 |  |  |  |
| France | **0.5** | **7** | 15.4 | 5.4 | 2.00 | 0.90 | 4.46 | **4** | 8.8 | 2.6 |  |  |  | **1** | 2.2 | 0.4 |  |  |  | **4** | 8.8 | 4.8 | 1.22 | 0.46 | 3.24 |
| For. Yugoslavia | **6.9** | **29** | 4.2 | 1.9 | 0.77 | 0.53 | 1.12 | **63** | 9.1 | 4.2 | 0.78 | 0.61 | 1.00 | **5** | 0.7 | 0.3 | 0.60 | 0.25 | 1.45 | **48** | 7.0 | 3.5 | 1.06 | 0.79 | 1.43 |
| Bosnian-Hercegovina | **5.0** | **20** | 4.0 | 2.1 | 0.95 | 0.61 | 1.49 | **54** | 10.9 | 6.3 | 1.25 | 0.95 | 1.65 | **4** | 0.8 | 0.4 |  |  |  | **20** | 4.0 | 2.2 | 0.84 | 0.54 | 1.31 |
| Italy | **0.5** | **5** | 11.0 | 2.6 | 1.18 | 0.49 | 2.83 | **6** | 13.2 | 4.3 | 0.75 | 0.34 | 1.66 | **0** |  |  |  |  |  | **4** | 8.8 | 3.0 |  |  |  |
| Spain | **0.4** | **2** | 4.5 | 2.5 |  |  |  | **4** | 8.9 | 4.7 | 0.66 | 0.25 | 1.75 | **0** |  |  |  |  |  | **3** | 6.7 | 3.5 |  |  |  |
| Greece | **0.9** | **9** | 9.8 | 3.9 | 1.50 | 0.78 | 2.88 | **9** | 9.8 | 4.0 | 0.67 | 0.35 | 1.29 | **0** |  |  |  |  |  | **3** | 3.3 | 1.2 |  |  |  |
| Croatia | **0.6** | **4** | 6.9 | 2.9 | 1.32 | 0.50 | 3.52 | **5** | 8.6 | 4.1 | 0.74 | 0.31 | 1.78 | **2** | 3.4 | 1.5 |  |  |  | **4** | 6.9 | 2.9 |  |  |  |
| Poland | **6.7** | **44** | 6.6 | 2.7 | 1.04 | 0.77 | 1.40 | **76** | 11.3 | 5.1 | 0.85 | 0.68 | 1.07 | **9** | 1.3 | 0.6 | 0.81 | 0.40 | 1.62 | **48** | 7.2 | 3.5 | 1.02 | 0.76 | 1.35 |
| Hungary | **1.6** | **19** | 11.8 | 2.4 | 1.24 | 0.79 | 1.95 | **30** | 18.7 | 5.4 | 0.96 | 0.67 | 1.38 | **3** | 1.9 | 0.3 |  |  |  | **23** | 14.3 | 4.4 | 1.49 | 0.99 | 2.24 |
| For. Czechoslovakia | **0.8** | **13** | 15.3 | 4.8 | 1.04 | 0.56 | 1.93 | **24** | 28.3 | 7.6 | 1.27 | 0.85 | 1.90 | **1** | 1.2 | 0.3 |  |  |  | **6** | 7.1 | 1.8 | 0.66 | 0.30 | 1.46 |
| Romania | **1.6** | **14** | 8.7 | 3.9 | 1.52 | 0.90 | 2.57 | **13** | 8.1 | 4.0 | 0.68 | 0.40 | 1.17 | **0** |  |  |  |  |  | **6** | 3.7 | 1.6 | 0.48 | 0.20 | 1.16 |
| For. Sovjetunionen | **1.0** | **14** | 14.0 | 3.4 | 1.18 | 0.70 | 2.00 | **20** | 20.0 | 7.5 | 0.90 | 0.58 | 1.42 | **2** | 2.0 | 0.1 |  |  |  | **10** | 10.0 | 2.5 | 1.00 | 0.54 | 1.85 |
| Russia | **1.2** | **1** | 0.9 | 0.6 |  |  |  | **11** | 9.5 | 6.1 | 1.33 | 0.73 | 2.41 | **1** | 0.9 | 0.4 |  |  |  | **4** | 3.5 | 2.5 | 0.74 | 0.28 | 1.98 |
| Estonia | **1.3** | **30** | 22.4 | 2.5 | 1.22 | 0.85 | 1.76 | **36** | 26.9 | 5.6 | 0.93 | 0.67 | 1.30 | **11** | 8.2 | 1.9 | 1.71 | 0.95 | 3.12 | **19** | 14.2 | 1.8 | 1.01 | 0.64 | 1.61 |
| UK & North Ireland | **1.0** | **3** | 2.9 | 0.7 |  |  |  | **17** | 16.3 | 7.4 | 1.14 | 0.71 | 1.83 | **1** | 1.0 | 0.5 |  |  |  | **9** | 8.6 | 4.5 | 1.13 | 0.59 | 2.17 |
| **All Non-NORDIC EUROPÉ** | **43.0** | **314** | 7.3 | 2.4 | 0.98 | 0.87 | 1.10 | **592** | 13.8 | 5.5 | 0.93 | 0.86 | 1.02 | **61** | 1.4 | 0.5 | 0.81 | 0.63 | 1.06 | **296** | 6.9 | 3.0 | 0.90 | 0.80 | 1.01 |
| USA | **1.3** | **16** | 12.6 | 2.9 | 1.17 | 0.72 | 1.91 | **19** | 14.9 | 5.1 | 0.74 | 0.47 | 1.18 | **6** | 4.7 | 0.4 | 1.34 | 0.56 | 3.22 | **9** | 7.1 | 3.8 | 0.74 | 0.39 | 1.42 |
| **All North AMERICA** | **2.3** | **18** | 7.9 | 2.6 | 1.04 | 0.66 | 1.65 | **21** | 9.2 | 4.2 | 0.62 | 0.40 | 0.96 | **6** | 2.6 | 0.4 | 1.09 | 0.45 | 2.61 | **13** | 5.7 | 3.1 | 0.76 | 0.44 | 1.31 |
| Chile | **2.8** | **8** | 2.9 | 1.5 | 0.62 | 0.31 | 1.24 | **12** | 4.3 | 2.8 | 0.42 | 0.24 | 0.74 | **1** | 0.4 | 0.1 |  |  |  | **6** | 2.1 | 1.4 | 0.38 | 0.17 | 0.84 |
| **All South AMERICA** | **5.3** | **11** | 2.1 | 1.2 | 0.52 | 0.29 | 0.95 | **22** | 4.1 | 2.8 | 0.44 | 0.29 | 0.67 | **1** | 0.2 | 0.1 |  |  |  | **9** | 1.7 | 1.1 | 0.33 | 0.17 | 0.63 |
| Ethiopia | **1.1** | **3** | 2.8 | 5.9 |  |  |  | **4** | 3.7 | 4.9 |  |  |  | **0** |  |  |  |  |  | **2** | 1.8 | 2.8 |  |  |  |
| Somalia | **1.8** | **4** | 2.3 | 4.1 |  |  |  | **1** | 0.6 | 0.8 |  |  |  | **0** |  |  |  |  |  | **3** | 1.7 | 1.2 |  |  |  |
| **All AFRICA** | **6.5** | **11** | 1.7 | 2.7 | 0.85 | 0.46 | 1.59 | **19** | 2.9 | 3.4 | 0.68 | 0.43 | 1.07 | **0** |  |  |  |  |  | **8** | 1.2 | 1.2 | 0.40 | 0.20 | 0.80 |
| Iraq | **5.6** | **9** | 1.6 | 1.6 | 0.64 | 0.33 | 1.24 | **21** | 3.8 | 3.5 | 0.65 | 0.42 | 1.01 | **0** |  |  |  |  |  | **10** | 1.8 | 1.7 | 0.46 | 0.25 | 0.86 |
| Syria | **1.9** | **4** | 2.1 | 1.8 |  |  |  | **6** | 3.2 | 2.6 | 0.47 | 0.21 | 1.05 | **1** | 0.5 | 0.4 |  |  |  | **4** | 2.1 | 1.7 |  |  |  |
| Lebanon | **1.9** | **5** | 2.7 | 3.8 | 1.20 | 0.50 | 2.89 | **6** | 3.2 | 2.5 | 0.57 | 0.26 | 1.28 | **0** |  |  |  |  |  | **2** | 1.1 | 0.5 |  |  |  |
| Iran | **5.2** | **9** | 1.7 | 1.2 | 0.50 | 0.26 | 0.96 | **21** | 4.1 | 2.9 | 0.51 | 0.33 | 0.78 | **0** |  |  |  |  |  | **15** | 2.9 | 1.9 | 0.61 | 0.37 | 1.02 |
| **All Middle EAST** | **14.5** | **27** | 1.9 | 1.7 | 0.65 | 0.44 | 0.95 | **54** | 3.7 | 3.0 | 0.56 | 0.43 | 0.73 | **1** | 0.1 | 0.0 |  |  |  | **31** | 2.1 | 1.7 | 0.51 | 0.35 | 0.72 |
| India | **1.2** | **3** | 2.5 | 3.8 |  |  |  | **2** | 1.6 | 1.3 |  |  |  | **0** |  |  |  |  |  | **1** | 0.8 | 0.9 |  |  |  |
| Vietnam | **1.2** | **3** | 2.5 | 2.2 |  |  |  | **0** |  |  |  |  |  | **2** | 1.6 | 1.2 |  |  |  | **2** | 1.6 | 1.4 |  |  |  |
| Thailand | **2.7** | **2** | 0.7 | 1.6 |  |  |  | **8** | 3.0 | 4.2 | 0.63 | 0.32 | 1.27 | **0** |  |  |  |  |  | **2** | 0.7 | 0.4 |  |  |  |
| China | **1.3** | **1** | 0.8 | 0.7 |  |  |  | **6** | 4.8 | 3.8 | 0.56 | 0.25 | 1.25 | **0** |  |  |  |  |  | **3** | 2.4 | 1.2 |  |  |  |
| **All ASIA** | **12.5** | **17** | 1.4 | 1.6 | 0.60 | 0.37 | 0.97 | **42** | 3.4 | 3.5 | 0.59 | 0.44 | 0.80 | **3** | 0.2 | 0.2 |  |  |  | **19** | 1.5 | 1.2 | 0.39 | 0.25 | 0.61 |

…continued (sTable2)

| Countries/region | **Malignant Melanoma** | | | | | | | **Mesothelioma** | | | | | | **Hematological** | | | | | | **Gall-bladder** | | | | | |
| --- | --- | --- | --- | --- | --- | --- | --- | --- | --- | --- | --- | --- | --- | --- | --- | --- | --- | --- | --- | --- | --- | --- | --- | --- | --- |
|  | Person years in 100000 | Total deaths | CMR per 100,000 | ASMR per 100,000 | Hazard ratio, 95% CI | | | Total deaths | CMR per 100,000 | ASMR per 100,000 | Hazard ratio, 95% CI | | | Total deaths | CMR per 100,000 | ASMR per 100,000 | Hazard ratio, 95% CI | | | **Total deaths** | CMR per 100,000 | ASMR per 100,000 | Hazard ratio, 95% CI | | |
| **SWEDEN (ref)** | **737.7** | **3,946** | 5.3 | 1.8 | 1.00 |  |  | **347** | 0.5 | 0.1 | 1.00 |  |  | **17,330** | 23.5 | 5.7 | 1.00 |  |  | **4,612** | 6.3 | 1.6 | 1.00 |  |  |
| **All IMMIGRANTS** | **118.1** | **324** | 2.7 | 1.1 | 0.64 | 0.57 | 0.72 | **67** | 0.6 | 0.2 | 1.44 | 1.10 | 1.90 | **1,950** | 16.5 | 5.7 | 1.02 | 0.97 | 1.07 | **399** | 3.4 | 1.2 | 0.78 | 0.70 | 0.87 |
| Finland | **24.4** | **115** | 4.7 | 1.4 | 0.77 | 0.63 | 0.92 | **19** | 0.8 | 0.2 | 1.28 | 0.81 | 2.04 | **619** | 25.4 | 6.4 | 1.03 | 0.95 | 1.11 | **151** | 6.2 | 1.5 | 0.91 | 0.77 | 1.08 |
| Denmark | **4.0** | **35** | 8.8 | 2.3 | 1.30 | 0.93 | 1.81 | **8** | 2.0 | 0.5 | 2.96 | 1.47 | 5.98 | **132** | 33.2 | 6.5 | 1.02 | 0.85 | 1.21 | **28** | 7.0 | 1.5 | 0.86 | 0.59 | 1.25 |
| Norway | **5.2** | **44** | 8.5 | 1.8 | 1.12 | 0.83 | 1.52 | **5** | 1.0 | 0.1 | 1.37 | 0.56 | 3.31 | **194** | 37.6 | 5.6 | 1.04 | 0.91 | 1.20 | **43** | 8.3 | 1.4 | 0.87 | 0.65 | 1.18 |
| **All NORDIC** | **33.8** | **197** | 5.8 | 1.6 | 0.91 | 0.78 | 1.05 | **32** | 0.9 | 0.2 | 1.50 | 1.04 | 2.16 | **949** | 28.1 | 6.3 | 1.03 | 0.96 | 1.10 | **222** | 6.6 | 1.5 | 0.89 | 0.78 | 1.03 |
| Turkey | **3.5** | **3** | 0.8 | 0.4 |  |  |  | **10** | 2.8 | 2.0 | 11.92 | 6.16 | 23.06 | **30** | 8.5 | 5.1 | 0.88 | 0.61 | 1.26 | **5** | 1.4 | 0.7 | 0.40 | 0.15 | 1.08 |
| Germany | **4.8** | **22** | 4.6 | 0.8 | 0.59 | 0.38 | 0.90 | **5** | 1.0 | 0.1 | 1.35 | 0.56 | 3.26 | **173** | 36.1 | 6.0 | 1.02 | 0.87 | 1.19 | **24** | 5.0 | 0.8 | 0.58 | 0.39 | 0.88 |
| Austria | **0.6** | **2** | 3.4 | 0.8 |  |  |  | **0** |  |  |  |  |  | **18** | 30.5 | 3.2 | 0.92 | 0.57 | 1.49 | **4** | 6.8 | 1.0 | 0.89 | 0.34 | 2.38 |
| Netherlands | **0.4** | **2** | 4.5 | 1.6 |  |  |  | **0** |  |  |  |  |  | **12** | 26.9 | 8.6 | 1.26 | 0.72 | 2.22 | **1** | 2.2 | 1.2 |  |  |  |
| France | **0.5** | **0** |  |  |  |  |  | **0** |  |  |  |  |  | **6** | 13.2 | 4.5 | 0.85 | 0.38 | 1.89 | **1** | 2.2 | 1.1 |  |  |  |
| For. Yugoslavia | **6.9** | **11** | 1.6 | 0.8 | 0.44 | 0.24 | 0.80 | **0** |  |  |  |  |  | **90** | 13.1 | 5.9 | 1.18 | 0.96 | 1.46 | **15** | 2.2 | 1.0 | 0.66 | 0.39 | 1.12 |
| Bosnian-Hercegovina | **5.0** | **16** | 3.2 | 1.8 | 1.11 | 0.67 | 1.83 | **2** | 0.4 | 0.2 |  |  |  | **54** | 10.9 | 5.7 | 1.34 | 1.02 | 1.76 | **14** | 2.8 | 1.4 | 1.19 | 0.70 | 2.03 |
| Italy | **0.5** | **1** | 2.2 | 1.1 |  |  |  | **1** | 2.2 | 0.3 |  |  |  | **15** | 33.1 | 10.3 | 1.45 | 0.86 | 2.44 | **2** | 4.4 | 0.6 |  |  |  |
| Spain | **0.4** | **3** | 6.7 | 2.6 |  |  |  | **0** |  |  |  |  |  | **7** | 15.6 | 4.9 | 1.10 | 0.52 | 2.30 | **0** | 0.0 |  |  |  |  |
| Greece | **0.9** | **0** |  |  |  |  |  | **1** | 1.1 | 0.4 |  |  |  | **9** | 9.8 | 3.6 | 0.71 | 0.37 | 1.37 | **2** | 2.2 | 0.9 |  |  |  |
| Croatia | **0.6** | **1** | 1.7 | 0.7 |  |  |  | **0** |  |  |  |  |  | **5** | 8.6 | 4.0 | 0.82 | 0.34 | 1.97 | **2** | 3.4 | 1.5 |  |  |  |
| Poland | **6.7** | **11** | 1.6 | 0.7 | 0.40 | 0.22 | 0.73 | **2** | 0.3 | 0.1 |  |  |  | **107** | 16.0 | 6.2 | 1.12 | 0.92 | 1.36 | **14** | 2.1 | 0.8 | 0.55 | 0.32 | 0.95 |
| Hungary | **1.6** | **5** | 3.1 | 0.6 | 0.56 | 0.23 | 1.35 | **1** | 0.6 | 0.2 |  |  |  | **26** | 16.2 | 4.0 | 0.71 | 0.48 | 1.05 | **5** | 3.1 | 0.8 | 0.57 | 0.24 | 1.36 |
| For. Czechoslovakia | **0.8** | **3** | 3.5 | 0.4 |  |  |  | **0** |  |  |  |  |  | **25** | 29.5 | 6.3 | 1.10 | 0.74 | 1.63 | **4** | 4.7 | 1.0 |  |  |  |
| Romania | **1.6** | **2** | 1.2 | 0.7 |  |  |  | **0** |  |  |  |  |  | **18** | 11.2 | 4.7 | 0.89 | 0.56 | 1.41 | **4** | 2.5 | 1.0 |  |  |  |
| For. Sovjetunionen | **1.0** | **2** | 2.0 | 0.1 |  |  |  | **2** | 2.0 | 0.2 |  |  |  | **25** | 25.1 | 4.6 | 0.81 | 0.54 | 1.22 | **3** | 3.0 | 0.4 |  |  |  |
| Russia | **1.2** | **1** | 0.9 | 0.5 |  |  |  | **0** |  |  |  |  |  | **5** | 4.3 | 3.0 | 0.67 | 0.28 | 1.60 | **0** | 0.0 |  |  |  |  |
| Estonia | **1.3** | **12** | 9.0 | 0.6 | 0.97 | 0.54 | 1.76 | **1** | 0.7 | 0.0 |  |  |  | **67** | 50.0 | 7.0 | 1.09 | 0.85 | 1.39 | **15** | 11.2 | 1.4 | 0.89 | 0.52 | 1.53 |
| UK & North Ireland | **1.0** | **5** | 4.8 | 1.5 | 1.07 | 0.45 | 2.58 | **0** |  |  |  |  |  | **15** | 14.4 | 4.8 | 0.96 | 0.58 | 1.58 | **0** | 0.0 |  |  |  |  |
| **All Non-NORDIC EUROPÉ** | **43.0** | **108** | 2.5 | 0.9 | 0.56 | 0.46 | 0.68 | **28** | 0.7 | 0.3 | 1.59 | 1.07 | 2.38 | **741** | 17.2 | 5.6 | 1.03 | 0.95 | 1.11 | **121** | 2.8 | 0.9 | 0.64 | 0.53 | 0.77 |
| USA | **1.3** | **6** | 4.7 | 1.5 | 0.80 | 0.36 | 1.79 | **0** |  |  |  |  |  | **36** | 28.2 | 5.2 | 0.93 | 0.66 | 1.32 | **7** | 5.5 | 1.0 | 0.83 | 0.40 | 1.75 |
| **All North AMERICA** | **2.3** | **6** | 2.6 | 0.9 | 0.58 | 0.26 | 1.29 | **0** |  |  |  |  |  | **39** | 17.1 | 4.7 | 0.83 | 0.59 | 1.15 | **11** | 4.8 | 2.1 | 0.96 | 0.52 | 1.79 |
| Chile | **2.8** | **3** | 1.1 | 0.6 |  |  |  | **0** |  |  |  |  |  | **24** | 8.6 | 5.0 | 0.88 | 0.59 | 1.32 | **12** | 4.3 | 2.5 | 1.50 | 0.83 | 2.72 |
| **All South AMERICA** | **5.3** | **4** | 0.7 | 0.4 |  |  |  | **0** |  |  |  |  |  | **38** | 7.1 | 4.8 | 0.84 | 0.61 | 1.16 | **16** | 3.0 | 2.0 | 1.27 | 0.77 | 2.12 |
| Ethiopia | **1.1** | **0** |  |  |  |  |  | **0** |  |  |  |  |  | **6** | 5.5 | 6.8 | 1.54 | 0.69 | 3.43 | **3** | 2.8 | 4.0 |  |  |  |
| Somalia | **1.8** | **0** |  |  |  |  |  | **0** |  |  |  |  |  | **8** | 4.6 | 6.1 | 1.16 | 0.58 | 2.33 | **0** | 0.0 |  |  |  |  |
| **All AFRICA** | **6.5** | **0** |  |  |  |  |  | **1** | 0.2 | 0.1 |  |  |  | **40** | 6.2 | 7.5 | 1.47 | 1.07 | 2.02 | **5** | 0.8 | 1.0 | 0.78 | 0.32 | 1.87 |
| Iraq | **5.6** | **2** | 0.4 | 0.4 |  |  |  | **2** | 0.4 | 0.5 |  |  |  | **27** | 4.8 | 4.3 | 0.94 | 0.64 | 1.38 | **3** | 0.5 | 0.6 |  |  |  |
| Syria | **1.9** | **1** | 0.5 | 0.3 |  |  |  | **0** |  |  |  |  |  | **10** | 5.4 | 3.9 | 0.85 | 0.46 | 1.58 | **2** | 1.1 | 1.1 |  |  |  |
| Lebanon | **1.9** | **0** |  |  |  |  |  | **0** |  |  |  |  |  | **5** | 2.7 | 3.6 | 0.54 | 0.23 | 1.30 | **0** | 0.0 |  |  |  |  |
| Iran | **5.2** | **2** | 0.4 | 0.2 |  |  |  | **2** | 0.4 | 0.2 |  |  |  | **39** | 7.5 | 5.3 | 0.99 | 0.72 | 1.35 | **7** | 1.4 | 1.0 | 0.66 | 0.32 | 1.39 |
| **All Middle EAST** | **14.5** | **5** | 0.3 | 0.3 | 0.13 | 0.06 | 0.32 | **4** | 0.3 | 0.3 |  |  |  | **81** | 5.6 | 4.6 | 0.91 | 0.73 | 1.13 | **12** | 0.8 | 0.8 | 0.51 | 0.29 | 0.90 |
| India | **1.2** | **2** | 1.6 | 2.2 |  |  |  | **0** |  |  |  |  |  | **5** | 4.1 | 6.1 | 0.84 | 0.35 | 2.03 | **1** | 0.8 | 1.0 |  |  |  |
| Vietnam | **1.2** | **1** | 0.8 | 1.0 |  |  |  | **0** |  |  |  |  |  | **6** | 4.9 | 4.7 | 0.77 | 0.34 | 1.70 | **1** | 0.8 | 1.0 |  |  |  |
| Thailand | **2.7** | **0** |  |  |  |  |  | **0** |  |  |  |  |  | **8** | 3.0 | 3.0 | 0.88 | 0.44 | 1.77 | **2** | 0.7 | 0.4 |  |  |  |
| China | **1.3** | **0** |  |  |  |  |  | **0** |  |  |  |  |  | **10** | 7.9 | 3.1 | 0.84 | 0.45 | 1.56 | **2** | 1.6 | 1.0 |  |  |  |
| **All ASIA** | **12.5** | **4** | 0.3 | 0.4 |  |  |  | **2** | 0.2 | 0.2 | 1.02 | 0.25 | 4.18 | **59** | 4.7 | 5.5 | 0.91 | 0.70 | 1.18 | **12** | 1.0 | 1.1 | 0.70 | 0.39 | 1.27 |

…continued (sTable2)

| Countries/region | **Head and Neck** | | | | | | |
| --- | --- | --- | --- | --- | --- | --- | --- |
|  | Person years in 100,000 | Total deaths | CMR per 100,000 | ASMR per 100,000 | Hazard ratio, 95% CI | | |
| **SWEDEN (ref)** | **737.7** | **2,049** | 2.8 | 0.8 | 1.00 |  |  |
| **All IMMIGRANTS** | **118.1** | **238** | 2.0 | 0.8 | 0.87 | 0.76 | 1.00 |
| Finland | **24.4** | **87** | 3.6 | 0.9 | 0.99 | 0.79 | 1.24 |
| Denmark | **4.0** | **18** | 4.5 | 0.6 | 1.15 | 0.72 | 1.83 |
| Norway | **5.2** | **25** | 4.8 | 1.2 | 1.10 | 0.74 | 1.63 |
| **All NORDIC** | **33.8** | **130** | 3.8 | 0.9 | 1.03 | 0.86 | 1.23 |
| Turkey | **3.5** | **4** | 1.1 | 0.9 |  |  |  |
| Germany | **4.8** | **12** | 2.5 | 0.3 | 0.60 | 0.34 | 1.06 |
| Austria | **0.6** | **6** | 10.2 | 1.6 | 2.67 | 1.20 | 5.95 |
| Netherlands | **0.4** | **1** | 2.2 | 1.1 |  |  |  |
| France | **0.5** | **0** |  |  |  |  |  |
| For. Yugoslavia | **6.9** | **4** | 0.6 | 0.2 |  |  |  |
| Bosnian-Hercegovina | **5.0** | **5** | 1.0 | 0.5 | 0.72 | 0.30 | 1.74 |
| Italy | **0.5** | **0** |  |  |  |  |  |
| Spain | **0.4** | **1** | 2.2 | 1.2 |  |  |  |
| Greece | **0.9** | **1** | 1.1 | 0.3 |  |  |  |
| Croatia | **0.6** | **1** | 1.7 | 0.8 |  |  |  |
| Poland | **6.7** | **6** | 0.9 | 0.4 | 0.41 | 0.18 | 0.91 |
| Hungary | **1.6** | **5** | 3.1 | 0.9 | 1.04 | 0.43 | 2.49 |
| For. Czechoslovakia | **0.8** | **4** | 4.7 | 0.8 |  |  |  |
| Romania | **1.6** | **4** | 2.5 | 1.6 |  |  |  |
| For. Sovjetunionen | **1.0** | **4** | 4.0 | 0.5 |  |  |  |
| Russia | **1.2** | **2** | 1.7 | 1.8 |  |  |  |
| Estonia | **1.3** | **2** | 1.5 | 0.1 |  |  |  |
| UK & North Ireland | **1.0** | **2** | 1.9 | 0.8 |  |  |  |
| **All Non-NORDIC EUROPÉ** | **43.0** | **67** | 1.6 | 0.5 | 0.67 | 0.52 | 0.86 |
| USA | **1.3** | **6** | 4.7 | 1.5 | 1.50 | 0.67 | 3.35 |
| **All North AMERICA** | **2.3** | **7** | 3.1 | 1.5 | 1.32 | 0.63 | 2.77 |
| Chile | **2.8** | **2** | 0.7 | 0.4 |  |  |  |
| **All South AMERICA** | **5.3** | **6** | 1.1 | 0.7 | 0.77 | 0.35 | 1.72 |
| Ethiopia | **1.1** | **0** |  |  |  |  |  |
| Somalia | **1.8** | **7** | 4.0 | 3.8 | 5.40 | 2.52 | 11.55 |
| **All AFRICA** | **6.5** | **9** | 1.4 | 1.4 | 1.80 | 0.93 | 3.50 |
| Iraq | **5.6** | **2** | 0.4 | 0.4 |  |  |  |
| Syria | **1.9** | **1** | 0.5 | 0.6 |  |  |  |
| Lebanon | **1.9** | **0** |  |  |  |  |  |
| Iran | **5.2** | **6** | 1.2 | 0.8 | 0.88 | 0.40 | 1.98 |
| **All Middle EAST** | **14.5** | **9** | 0.6 | 0.5 | 0.55 | 0.28 | 1.06 |
| India | **1.2** | **0** |  |  |  |  |  |
| Vietnam | **1.2** | **1** | 0.8 | 0.5 |  |  |  |
| Thailand | **2.7** | **4** | 1.5 | 0.6 |  |  |  |
| China | **1.3** | **1** | 0.8 | 0.7 |  |  |  |
| **All ASIA** | **12.5** | **10** | 0.8 | 0.7 | 0.85 | 0.45 | 1.59 |
